# Influence of Switchgrass *TDIF-like* Genes on *Arabidopsis* Vascular Development

**DOI:** 10.3389/fpls.2021.737219

**Published:** 2021-09-23

**Authors:** Dongdong Tian, Jingwen Tang, Liwen Luo, Zhe Zhang, Kebing Du, Robert M. Larkin, Xueping Shi, Bo Zheng

**Affiliations:** ^1^Key Laboratory of Horticultural Plant Biology of Ministry of Education, College of Horticulture and Forestry Sciences, Huazhong Agricultural University, Wuhan, China; ^2^Tobacco Research Institute, Chinese Academy of Agricultural Science, Qingdao, China; ^3^Hubei Engineering Technology Research Center for Forestry Information, Huazhong Agricultural University, Wuhan, China

**Keywords:** switchgrass, CLE, TDIF, vascular development, biomass

## Abstract

As a member of the CLAVATA3 (CLV3)/EMBRYO SURROUNDING REGION (CLE) family, the dodecapeptide tracheary element differentiation inhibitory factor (TDIF) has a major impact on vascular development in plants. However, the influence of polymorphisms in the TDIF peptide motif on activity remains poorly understood. The model plant, *Arabidopsis* provides a fast and effective tool for assaying the activity of *TDIF* homologs. Five *TDIF* homologs from a group of 93 *CLE* genes in switchgrass (*Panicum virgatum*), a perennial biomass crop, named *PvTDIF-like* (*PvTDIFL*) genes were studied. The expression levels of *PvTDIFL1, PvTDIFL3*^*MR*3^, and *PvTDIFL3*^*MR*2^ were relatively high and all of them were expressed at the highest levels in the rachis of switchgrass. The precursor proteins for PvTDIFL1, PvTDIFL3^MR3^, and PvTDIFL3^MR2^ contained one, three, and two TDIFL motifs, respectively. Treatments with exogenous PvTDIFL peptides increased the number of stele cells in the hypocotyls of *Arabidopsis* seedlings, with the exception of PvTDIFL_4p. Heterologous expression of *PvTDIFL1* in *Arabidopsis* strongly inhibited plant growth, increased cell division in the vascular tissue of the hypocotyl, and disrupted the cellular organization of the hypocotyl. Although heterologous expression of *PvTDIFL3*^*MR*3^ and *PvTDIFL3*^*MR*2^ also affected plant growth and vascular development, PvTDIFL activity was not enhanced by the multiple TDIFL motifs encoded by *PvTDIFL3*^*MR*3^ and *PvTDIFL3*^*MR*2^. These data indicate that in general, PvTDIFLs are functionally similar to *Arabidopsis* TDIF but that the processing and activities of the PvTDIFL peptides are more complex.

## Introduction

In plants, the *CLAVATA3 (CLV3)/EMBRYO SURROUNDING REGION (CLE)* gene family plays a vital role in cell division and cell differentiation by mediating intercellular communication (Fletcher et al., 1999; Fiers et al., 2005, 2007). *CLE* peptides are derived from a pre-propeptide containing an N-terminal signal peptide and a conserved C-terminal motif of 12-13 amino acids. A few *CLE* genes encode multiple CLE motifs in rice, wheat (*Triticum aetivum*), *Picea*, and *Amborella*, etc. (Goad et al., 2017). *CLE* genes are ubiquitous in the plant kingdom, and have been comprehensively studied in eudicots (*Arabidopsis thaliana, Glycine max, Lycopersicon esculentum, Gossypium hirsutum, Populus trichocarpa*, and *Vitis vinifera*), monocots (*Oryza sativa, T. aestivum*, and *Zea mays*), and gymnosperms (*Picea abies* and *P. glauca*) (Cock and McCormick, 2001; Sawa et al., 2008; Strabala et al., 2014; Han et al., 2016; Goad et al., 2017; Wang et al., 2019). In *A. thaliana*, at least 32 *CLE* genes have been identified (Ito et al., 2006; Kondo et al., 2006; Ohyama et al., 2009; Ogawa-Ohnishi et al., 2013). Based on their biological functions, the *Arabidopsis* CLE peptides were grouped into two major classes. The A-type CLE peptides which maintain the apical meristems in shoots and roots and include CLV3, CLE1-27, and CLE40. The B-type/H-type CLE peptides, on the other hand, suppress xylem differentiation and promote vascular procambial cell division and include CLE41, CLE42, and CLE44 (Schoof et al., 2000; Ito et al., 2006; Whitford et al., 2008; Stahl et al., 2009; Hirakawa et al., 2010a,b).

One member of the CLE family, a dodecapeptide (H-E-V-P-S-G-P-N-P-I-S-N) named tracheary element differentiation inhibitory factor (TDIF) was first identified in a *Zinnia elegans* mesophyll cell xylogenesis system. H^1^ is unique among CLE peptides with TDIF activity, and V^3^, N^8^, and N^12^ are essential for TDIF activity (Ito et al., 2006). In *Arabidopsis*, TDIF is encoded by two genes, *CLE41* and *CLE44*. *CLE42* and *CLE46* are classified as *TDIF*-*like* (*TDIFL*) genes, because their CLE motifs (H-G-V-P-S-G-P-N-P-I-S-N and H-K-H-P-S-G-P-N-P-T-G-N, respectively) are highly homologous to the TDIF motif. The *TDIF* genes are mainly involved in the regulation of vascular development. The TDIF signaling pathway in the vascular meristem has been well-studied (Ito et al., 2006; Ohyama et al., 2008). The TDIF RECEPTOR/PHLOEM INTERCALATED WITH XYLEM (TDR/PXY), a member of the leucine-rich repeat receptor-like kinase (LRR-RLK) family, is the TDIF receptor in procambial cells (Fisher and Turner, 2007; Hirakawa et al., 2008). The TDIF peptide that is produced in the phloem controls vascular procambial or cambial cell proliferation and xylem differentiation by activating TDR/PXY in the procambium or cambium. Two transcription factors, WUSCHEL HOMEOBOX RELATED 4 (WOX4) and WOX14, regulate the proliferation of plant vascular tissue by acting downstream of the TDIF–TDR/PXY signaling pathway (Hirakawa et al., 2010a,b; Etchells et al., 2013; Kucukoglu et al., 2017; Li et al., 2018). In addition, an NAC domain transcription factor, XVP, fine-tunes the TDIF signaling that contributes to vascular development by serving as a negative regulator (Kucukoglu, 2020; Yang et al., 2020). Current reports on TDIF/TDIFL have focused on genes encoding a single motif. Little is known about the function of TDIF/TDIFL genes encoding multiple motifs.

Switchgrass (*P. virgatum*) is a highly productive herbaceous perennial that thrives in diverse environments and is, therefore, a model perennial biomass crop (PBC) (Sanderson et al., 1996; McLaughlin and Adams Kszos, 2005). It has been a feedstock for the biofuels and specialty chemicals (Parrish and Fike, 2005; Sanderson et al., 2006; Keshwani and Cheng, 2009). Among the four PBCs (poplar, switchgrass, *Miscanthus*, and *Salix*), molecular regulation of lignocellulosic formation has been comprehensively studied in poplar trees (Clifton-Brown et al., 2019). Increasing vascular cambial activity results in incremental xylem and phloem, therefore increasing the biomass of wood. The division of vascular cambial cells was stimulated by manipulating CLE41–PXY signaling in hybrid poplar (Etchells and Turner, 2010; Etchells et al., 2015). The WOX4 controls the rate of cambial cell division and hence, the growth of stem girth in a TDIF-dependent manner (Kucukoglu et al., 2017). In a recent report, the *CLE* gene family in switchgrass, including three *PvTDIFL* genes, was defined. The statistical analysis demonstrated that no common TDIF/TDIFL motif is shared between monocots and dicots and that the contribution of TDIF/TDIFL to the development of vascular tissue has probably been diverged in monocots and dicots (Zhang et al., 2020). However, the knowledge of TDIF/TDIFL peptide functions in switchgrass remains limited.

Studying the mechanisms that drive rapid growth in switchgrass and other PBCs may help to introduce biomass-related genes, pathways or regulatory modules into dicotyledonous plants. *Arabidopsis* is widely used in the study of secondary cell wall formation and vascular development because although *Arabidopsis* is an annual herbaceous plant, it has most of the cell types associated with secondary growth (Zhang et al., 2011; Ursache et al., 2013; Ragni and Hardtke, 2014). The TDIF–TDR/PXY signaling pathway has been well-studied in *Arabidopsis* (Etchells and Turner, 2010; Hirakawa et al., 2010a,b; Etchells et al., 2013, 2016; Wang et al., 2013; Kondo and Fukuda, 2015; Zhang et al., 2016; Kucukoglu, 2020). Thus, *Arabidopsis* is suitable for activity screening and functional analysis of *TDIF*/*TDIFL* genes from various plants. This study aimed to use bioinformatics tools to identify the CLE gene family, including *TDIF*/*TDIFL* genes, at the whole genome level in *P. virgatum*. Treatment of *Arabidopsis* with exogenous TDIF/TDIFL peptides and heterologous expression of *TDIF*/*TDIFL* genes in *Arabidopsis* allowed the researchers of this study to test whether particular PvTDIFL peptides influenced plant growth and vascular development. Rapid characterization of PvTDIFL peptides in *Arabidopsis* can provide a preliminary understanding of the biological activities of PvTDIFL peptides and indicate potential biotechnological applications related to wood formation and biomass improvement. This is not practical to achieve with switchgrass because the genetic transformation of switchgrass is still time-consuming and laborious (Xi et al., 2009; Chen and Song, 2019; Ondzighi-Assoume et al., 2019).

## Materials and Methods

### Identification of *CLE* Genes in *P. virgatum*

The amino acid sequences of *Arabidopsis* CLE proteins were downloaded from The *Arabidopsis* Information Resource (TAIR) (https://www.Arabidopsis.org/). The 12-amino acid CLE motifs were used as queries in TBLASTN search of the *P. virgatum* v1.1 genome with a threshold e-value of 500 in the Phytozome v12.1 database (https://phytozome.jgi.doe.gov/pz/portal.html) (Goodstein et al., 2012). Repeated hits were removed and only one hit at each chromosomal location was kept. Genes at each chromosomal location were identified as potential *CLE* genes. If there was no annotated gene at the target location, a 5-kb genome fragment with the target location in the center was then retrieved and subjected to an analysis with the online software, FGENESH, for gene predictions on the softberry website (http://linux1.softberry.com/) (Solovyev et al., 2006). Amino acid sequences encoded by each of the potential *CLE* genes were checked for the C-terminal conserved CLE motifs. All of the newly identified motifs were used as queries in TBLASTN searches, as described in the preceding steps. The analysis was iterated until no more *CLE* candidate could be identified. All of the *CLE* candidates were compared with the previously reported *CLE* genes in *P. virgatum* (Zhang et al., 2020). The final *TDIF/TDIFL* gene sequences were obtained using cloning based on the nucleotide sequences from Phytozome (Supplementary Figure 1).

### Bioinformatics Analysis

To classify *PvCLE* genes, all 12-aa CLE motifs encoded by *AtCLE* and *PvCLE* genes were extracted, including the *PvCLE* genes encoding multiple CLE motifs. Phylogenetic trees containing the 12-aa CLE motif sequences were constructed as previously described (Zhang et al., 2020). Based on the clustering of CLE motifs, *PvCLE* genes in the same group as *AtTDIF*/*TDIFL* genes were predicted as *PvTDIFL* genes. To further analyze the evolutionary relationship between *PvTDIFL* and *AtTDIF*/*TDIFL* genes, phylogenetic trees containing the full-length amino acid sequences for TDIF/TDIFL from both *P. virgatum* and *Arabidopsis* were constructed using the MEGA 5.05 software (Hall, 2013) with the following parameters: alignment, Muscle, phylogeny construct or test, Maximum Likelihood Tree, and number of bootstrap replicatio*n* = 1000. Signal peptides that target the TDIF/TDIFL proteins to the secretary pathway were predicted with the SMART Server using a normal model (http://smart.embl-heidelberg.de/smart/set_mode.cgi?NORMAL=1) (Letunic and Bork, 2018). Gene structure analysis was performed using the Gene Structure Display Server 2.0 (http://gsds.cbi.pku.edu.cn/) (Hu et al., 2015). The Format of Gene Features was set as fast-all (FASTA) Sequence. Other features containing signal peptides and motifs were uploaded in Browser Extensible Data (BED) format. Phylogenetic Tree was uploaded in Newick format based on full-length amino acid sequences (Hu et al., 2015). Multiple alignments were performed using the DNAMAN v6.0 software (Lynnon Biosoft, Quebec, Canada). Weblogo-Create Sequence Logos (http://weblogo.berkeley.edu/logo.cgi) was used for comparative analysis of motif conservation and for conservation analysis of each amino acid site of TDIF/TDIFL motifs in *Arabidopsis* and switchgrass (Crooks et al., 2004). Isoelectric point (*p*I) and molecular weight (MW) were calculated using the ExPASy-ProtParam tool (http://web.expasy.org/protparam/) (Bjellqvist et al., 1993).

### Plant Materials and Growth Conditions

*Arabidopsis thaliana* ecotype Columbia-0 (Col-0) and *P. virgatum* were used in this study. *Arabidopsis* seeds were surface sterilized in 75% (v/v) ethanol for 1 min and 5% (v/v) sodium hypochlorite for 15 min, with occasional gentle shaking. The seeds were then washed five times with sterilized distilled H_2_O (dH_2_O). After stratification at 4°C for 2 days, the seeds were placed in liquid Murashige and Skoog (MS) medium (pH 5.8) containing 1.5% sucrose (w/v) or on solid MS medium (pH 5.8) containing 1.5% sucrose (w/v) and 0.7% plant agar (w/v). The conical flasks containing liquid medium and the plates containing solid medium were placed in a tissue culture room maintained at 23°C and grown in a photoperiod containing 16 h of light at a fluence rate of 100 μmol photons m^−2^ s^−1^ followed by 8 h of dark. The conical flasks were placed on an orbital shaker at a rotational speed of 80 rpm. The plates were positioned vertically. *Arabidopsis* were grown in soil under the same temperature and light conditions, except that light intensity was set as 150 μmol photons m^−2^ s^−1^. The *P. virgatum* plants used in this study were grown at Huazhong Agricultural University, Wuhan, China.

### Exogenous Peptide Treatment

The TDIF/TDIFL peptides derived from *Arabidopsis* TDIF and PvTDIFL motifs (Supplementary Table 1) were chemically synthesized by GenScript Biotech Corporation (Nanjing, China). Twenty milligrams of each peptide was provided at a purity ≥ 90% (w/w). Peptides were dissolved in double distilled water (ddH_2_O) at a stock concentration of 1 mg/ml and stored at −80°C for future use. Synthetic TDIF and PvTDIFL peptides were added to liquid medium at a final concentration of 10 μM as previously described (Whitford et al., 2008). *Arabidopsis* seeds were treated with 10 ml of medium in conical flasks on an orbital shaker at a rotational speed of 80 rpm for 10 days. Each treatment required six flasks, with six seeds per flask.

### RNA Extraction and qRT-PCR Analysis of *PvTDIFL* Genes

Five different tissues of *P. virgatum*, namely, seed (mature seeds), rachis (bearing mature seeds), leaf (fully expanded leaf), stem (middle internode of seedling), and root were sampled in triplicates and immediately frozen in liquid nitrogen. Total RNA was extracted using the 2 × CTAB method (Li et al., 2008). The pellet was dissolved in 30 μl of RNase free ddH_2_O. The concentration and quality of RNA were measured with a NanoDrop® 2000 spectrophotometer (Thermo Scientific, Wilmington, Delaware, USA). The RNA samples with A_260_/A_280_ ratios that ranged from 1.8 to 2.1 and A_260_/A_230_ ratios ≥2 were stored at −80°C for future use.

To quantify the relative expression levels of *PvTDIFL* genes in different samples, 1 μg of total RNA was used as a template to synthesize cDNA using the PrimeScript™ RT reagent Kit with gDNA Eraser (Perfect Real Time) (TaKaRa, Dalian, China). The qRT-PCR reactions were then prepared using 2 × HSYBR qPCR Mix without ROX (ZOMANBIO, Beijing, China). A standard 2-step amplification protocol was run in a LightCycler® 96 Real-Time PCR System (Roche, USA). The comparative C_T_ method (ΔΔCt method) was used for relative quantification of real-time PCR (Pfaffl, 2001). *PvUBQ6* (Pavir.5NG345900) was used as the reference gene (Gimeno et al., 2014). All gene specific primers (Supplementary Table 2) were designed using the Primer Premier 5.0 software (www.PremierBiosoft.com). All real-time PCR reactions were run in three biological replicates and two technical replicates.

### Plasmid Construction and Generation of Transgenic Plants

The coding sequences (CDS) from *PvTDIFL3*^*MR*3^ and *PvTDIFL3*^*MR*2^ were amplified using PCR with gene specific primers (Supplementary Table 2). The CDS of *PvTDIFL1* was artificially synthesized (Sunny Technology, Shanghai, China). The products were cloned into a Gateway™ entry vector *pDONR201* using the BP recombination reaction (Invitrogen). The clones were analyzed using DNA sequencing. The correct fragments were subsequently recombined into the destination vector, *pK2GW7* (Karimi et al., 2002). Transformation of *A. thaliana* was performed using the *Agrobacterium tumefaciens*-mediated floral dip method (Clough and Bent, 1998).

### Morphological and Histological Analysis of *Arabidopsis* Seedlings

*Arabidopsis* seedlings were photographed with a Canon EOS 7D digital camera to obtain high-resolution images. Root length was measured by using the ImageJ software (http://imagej.nih.gov/ij). The images presented in the figures were representative images from images of 24 individual seedlings for each line for the plate-grown plants and 10 individual seedlings for each line for the soil-grown plants, respectively.

Histological analysis of the vasculature was performed by using semi-thin sections. The upper part of the hypocotyl, 2–3 mm in length, was cut with a scalpel. A small amount of tissue from the stem and petiole of the rosette was kept to mark the upper end of the hypocotyl segment. The hypocotyl segments were fixed in formalin-acetic acid-alcohol (FAA) fixative (50% ethanol, 10% glacial acetic acid, 5% formaldehyde, v/v/v) at 4°C for 24 h. Dehydration was carried out by immersing the specimen in an ascending series of ethanol. Lastly, the specimens were infiltrated and embedded with Technovit® 7100 resin (www.kulzer-technik.com). The hypocotyl specimens were transversely sectioned at a thickness of 2.5 μm using a RM2265 Microtome (Leica BIOSYSTEMS, Nussloch, Germany) beginning from the upper end. The rosette tissues were first trimmed and monitored by sectioning and microscopy. The 10th to 20th sections of the hypocotyls were stained with 0.05% (w/v) aqueous toluidine blue and visualized with a BX53 light microscope (OLYMPUS, Tokyo, Japan). The cells in the stele were marked by using the ImageJ software and the number of cells was counted manually.

Statistical analysis was performed with six biological replicates and a *t*-test was used with double sample variance assumption. The letters a, b, and c and asterisks indicate statistically significant differences relative to the control.

### Accession Numbers

Sequence data from this article are from the *P. virgatum* v1.1 genome in the Phytozome v12.1 database and TAIR10 associated with the following accession numbers: *AtEF1-*α (At5G60390), *PvUBQ6* (Pavir.5NG345900), *AtCLE41* (AT3G24770), *AtCLE42* (AT2G34925), *AtCLE44* (AT4G13195), *AtCLE46* (AT5G59305), *PvTDIFL1* (Pavir.Ab03264), *PvTDIFL2* (Pavir.Aa00134), and *PvTDIFL4* (Pavir.J06189). The sequence data for PvTDIFL3^MR3^ and PvTDIFL3^MR2^ has been submitted to GenBank and are associated with accession numbers MZ463198 and MZ463199, respectively.

## Results

### Identification of *CLE* Genes in *P. virgatum*

**Figure 1:**
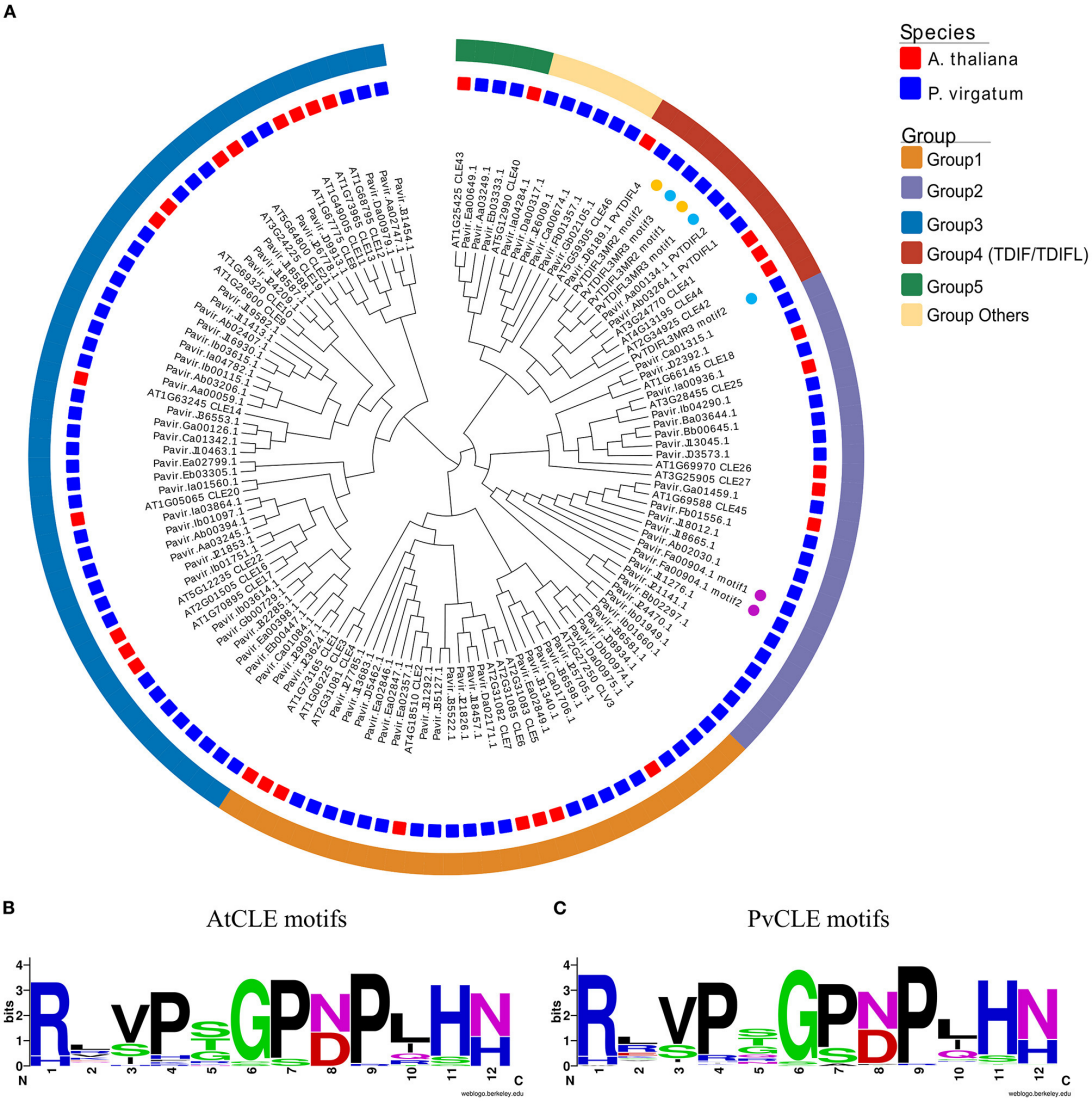
Phylogenetics and conservation analysis of *CLE* genes in *P. virgatum* and *A. thaliana*. **(A)** Phylogeny of 12-aa motif sequences from PvCLEs and AtCLEs. The phylogenetic tree was constructed as previously described (Zhang et al., 2020). Red and blue quadrates in the middle layer indicate AtCLEs and PvCLEs, respectively. Colored boxes in the outermost layer indicate different groups; colored dots in inner layer indicate genes with multiple motifs. **(B,C)** Weblogo images of CLE motifs from AtCLEs **(B)** and PvCLEs **(C)**, created by using the Weblogo online tool with the amino acid sequences from CLE peptides.

To identify *CLE* genes in *P. virgatum* (*PvCLE*), sequences similar to the *Arabidopsis* CLE motifs were identified in the *P. virgatum* v1.1 genome (https://phytozome.jgi.doe.gov/pz/portal.html). The *PvCLE* genes were predicted and clustered using the recently reported method (Zhang et al., 2020). In total, 93 *PvCLE* genes were identified, including 91 annotated *PvCLE*s in the *P. virgatum* v1.1 genome and two novel *PvCLE*s identified in this study (Figure 1, Supplementary Table 3). Since there were three *PvCLE*s that encoded multiple CLE motifs, all *PvCLE*s encoded a total of 97 CLE motifs. The total number of *PvCLE*s was nearly three times of the number of *AtCLE*s. Based on the clustering, *PvCLE*s were divided into six groups (Figure 1A, Supplementary Figure 2). The Weblogo of CLE motifs showed that Group 1, including eight *AtCLE*s (*AtCLE1-7*, and *AtCLV3*) and 21 *PvCLE*s, had two conserved amino acids, namely, Asp (D) at the 8th residue and His (H) at 12th residue. These two residues were replaced with Asn (N) in Group 3, which contained 13 *AtCLE*s (*AtCLE8-14, AtCLE16-17*, and *AtCLE19-22*) and 38 *PvCLE*s. Residue number 12 was Asn (N) in Group 2, which contained five *AtCLE*s (*AtCLE18, AtCLE25-27*, and *AtCLE45*) and 20 *PvCLE*s (Supplementary Figure 2). The Pavir.Fa00904.1 from Group 2 encoded two repeats of RRVRRGSDPIHN, therefore the Group 2 *PvCLE*s encoded a total of 21 CLE motifs. Group 4 was comprised of nine *TDIF*/*TDIFL* genes, with four members from *Arabidopsis* (*AtCLE41, 42, 44*, and *46*) and five genes from *P. virgatum*. In Group 4, there were two *PvCLE*s encoding multiple TDIFL motifs (see the next paragraph for details), and the total number of TDIFL motifs encoded in *P. virgatum* was as many as eight. Group 5 had two *Arabidopsis* members (*AtCLE40* and *AtCLE43*) and three *PvCLE*s. In general, the motif conservation in Group 5 was lower relative to groups 1 through 4. Six *PvCLE* genes were found upon encoding CLE motifs that are very different from the AtCLE motifs and thus, fell into “Group others” (Figure 1A, Supplementary Table 3). The CLE motifs are rather conserved between the AtCLEs and PvCLEs (Figures 1B,C).

**Figure 2:**
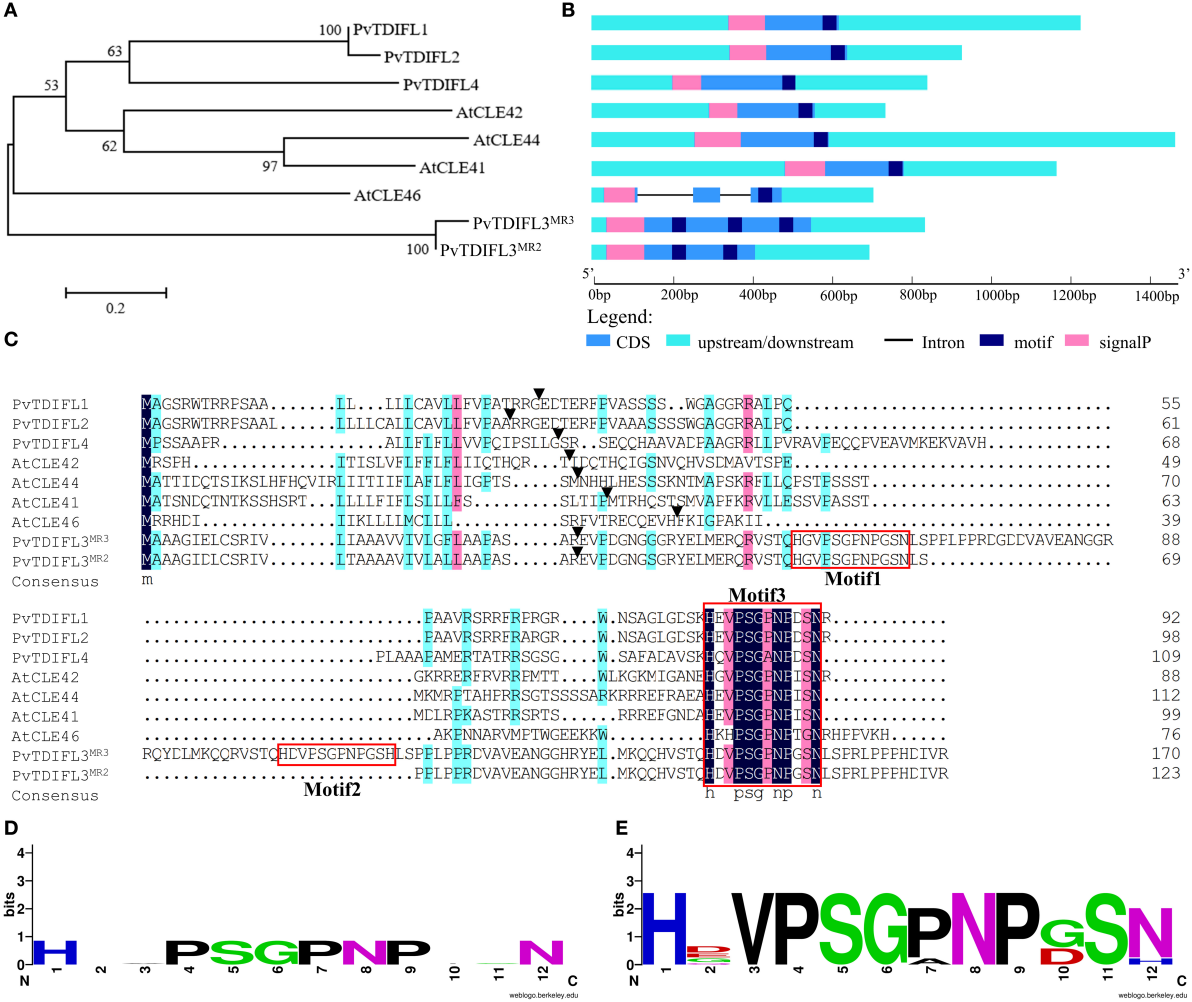
Phylogenetic analysis and genomic organization of tracheary element differentiation inhibitory factor (TDIF) in *P. virgatum* and *A. thaliana*. **(A)** Phylogeny of full-length protein sequences from PvTDIFLs and AtTDIF. The alignment was generated by using Muscle with the Maximum Likelihood method and 1,000 bootstrap iterations. Numbers on the nodes indicate clade credibility values. **(B)** Gene structures of *PvTDIFLs* and *AtTDIF* from nucleotide sequences. Gene structure analysis was performed using the gene structure display server 2.0. Cyan box, 3′ UTR and 5′ UTR; blue box, CDS; pink box, signal peptide; navy-blue box, TDIF peptide; black line, intron. **(C)** Multiple amino acid sequence alignment of PvTDIFL and AtTDIF proteins. Multiple alignments were performed using the DNAMAN v6.0 software. TDIF peptides are boxed in red. Black triangles indicate the SignalP cleavage site. **(D,E)** Weblogo images of AtTDIF **(D)** and PvTDIFL **(E)**. The images were created by using the Weblogo online tool with the amino acid sequences from AtTDIF and PvTDIFL peptides, respectively.

In total, five *TDIF* homologous genes were identified in the *P. virgatum* genome (Figure 2, Supplementary Figure 1, Table 1). The three *TDIF* homologs that were annotated are Pavir.Ab03264, Pavir.Aa00134, and Pavir.J06189, namely *PvTDIF-like1* (*PvTDIFL1*), *PvTDIFL2*, and *PvTDIFL4* in this study, respectively. A novel *TDIFL* gene, *PvTDIFL3*, was identified from TBLASTN searches of the *P. virgatum* v1.1 genome with a threshold e-value of 500. The predicted PvTDIFL3 protein product contained three potential TDIFL motifs and hence named PvTDIFL3^MR3^. The MR3 is referring to that it has three motif repeats (Figure 2, Table 1). When gene-specific primers were used to amplify the CDS of *PvTDIFL*s, a shorter fragment derived from *PvTDIFL3*^*MR*3^ was also cloned that was named *PvTDIFL3*^*MR*2^ (Figures 2A–C). The amino acid sequence of PvTDIFL3^MR2^ is identical to that of PvTDIFL3^MR3^, except that 47 amino acid residues are missing from the middle. The missing 47 amino acid residues constitute the second TDIFL motif and its flanking sequences of PvTDIFL3^MR3^ (Figure 2C). The *PvTDIFL3*^*MR*2^ matched to the same chromosomal location as *PvTDIFL3*^*MR*3^ in TBLASTN search of the *P. virgatum* v1.1 genome. In a phylogenetic analysis, amino acid sequences from the five *PvTDIFL* genes clustered in the same clade as the amino acid sequences from *Arabidopsis TDIF/TDIFL* genes (Figures 1, 2A). The nucleotide sequences were subjected to the online gene prediction program FGENESH on the Softberry website (http://linux1.softberry.com/), to evaluate the genomic sequence and gene structure of these five genes.

**Table 1:** Information of PvTDIFL peptides used in this study.

**Protein symbol**	**Gene ID**	**Protein length (AA)**	**SignalP cleavage site**	**Position of motif (AA)**	**PvTDIFL motif**
PvTDIFL1	Pavir.Ab03264	92	31	80-91	HEVPSGPNPDSN
PvTDIFL2	Pavir.Aa00134	98	31	86-97	HEVPSGPNPDSN
PvTDIFL3^MR3^	NA	171	32	56-67	HGVPSGPNPGSN
				103-114	HDVPSGPNPGSH
				146-157	HDVPSGPNPGSN
PvTDIFL3^MR2^	NA	124	32	56-67	HGVPSGPNPGSN
				99-110	HDVPSGPNPGSN
PvTDIFL4	Pavir.J06189	109	26	98-109	HQVPSGANPDSN

*NA, indicates no annotation*.

All the TDIF/TDIFL proteins from *Arabidopsis* and switchgrass contained a signal peptide for the secretory pathway at the N-terminus (Figures 2B,C). Similar to the *Arabidopsis* TDIF proteins, PvTDIFL1, 2, and 4 had a single TDIFL motif at the C-terminus. However, PvTDIFL3^MR3^ and PvTDIFL3^MR2^ had three and two TDIFL motifs, respectively (Figures 2B,C). The PvTDIFL proteins ranged from 92 to 175 amino acid residues in length. Their theoretical molecular weights (MW) and isoelectric points (*p*I) ranged from 10.01 to 18.50 kDa and from 5.81 to 11.94, respectively (Table 1).

A multiple sequence alignment analysis revealed that PvTDIFL1 and 2 proteins shared the same motif, HEVPSGPNPDSN, which is different from the *Arabidopsis* TDIF motif at the 10th residue in that an Ile (I) residue is changed to an Asp (D) residue. Motif 1 and motif 3 of PvTDIFL3^MR3^/PvTDIFL3^MR2^ were different at the 2nd and 10th residues relative to the TDIF motif. The 12th residue of Motif 2 from PvTDIFL3^MR3^ had an Asn (N) to His (H) substitution. The 2nd, 7th, and 10th residues of the motif from PvTDIFL4 were different from the TDIF motif (Figure 2C). The conserved TDIF/TDIFL dodecapeptides in *Arabidopsis* and switchgrass were compared by using a WebLogo analysis. Similar to the *Arabidopsis* TDIF, the 4th and 7th residues of the TDIFL motifs in PvTDIFL proteins were conserved Pro (P) residues. However, their 2nd, 10th and 12th residues were less conserved (Figures 2D,E).

### Tissue-Specific Expression Analysis of *PvTDIFL* Genes in *P. virgatum*

In *Arabidopsis, CLE41* is expressed in the phloem and the neighboring pericycle cells in the root and hypocotyl. *CLE44* is expressed in the phloem, pericycle and endodermal cells (Hirakawa et al., 2008). To analyze the expression pattern of *PvTDIFL* genes in the different tissues of switchgrass, *in silico* expression analysis and qRT-PCR were both performed.

**Figure 3:**
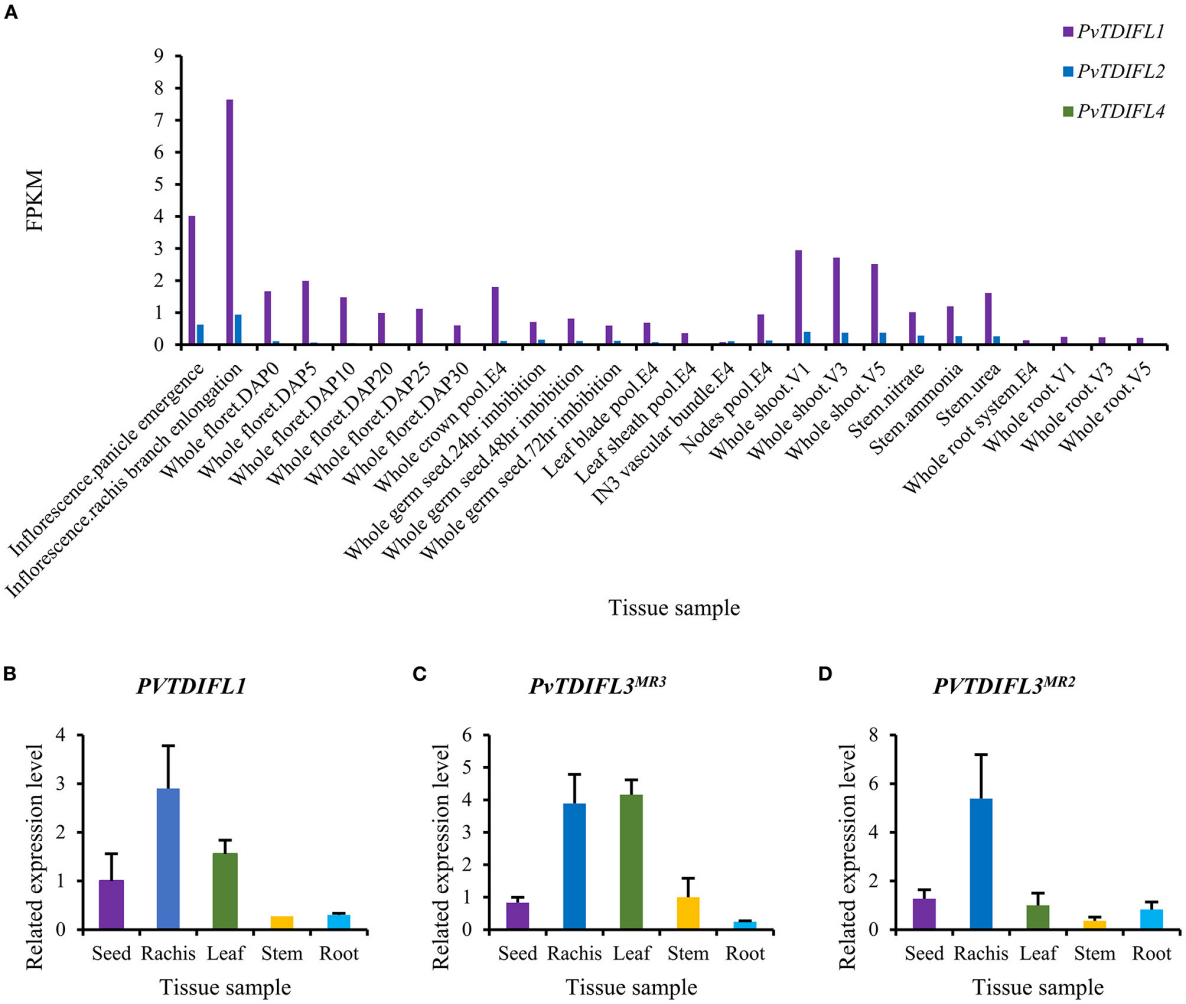
Expression analysis of *PvTDIFL* genes in *P. virgatum*. **(A)** Expression of *PvTDIFL1, 2* and *4* in different samples of *P. virgatum*. Fragments Per Kilobase per Million (FPKM) values of *PvTDIFL1, 2*, and *4* were obtained from *in silico* expression data in Phytozome. Samples listed are as follows: Inflorescence.Panicle emergence, Inflorescence.rachis branch elongation, Whole floret.DAP0, 5, 10, 20, 25, 30, Whole crown pool.E4, Whole germ seed 24 h imbibition, 48 h imbibition, 72 h imbibition, Leaf blade pool.E4, Leaf sheath pool.E4, IN3 vascular bundle.E4, Nodes pool.E4, Whole shoot.V1, 3, 5, Stem.nitrate, Stem.ammonia, Stem.urea, Whole root system.E4, Whole root.V1, 3, 5. **(B–D)** Relative expression levels of *PvTDIFL1* **(B)**, *PvTDIFL3*^*MR*3^ **(C)**, and *PvTDIFL3*^*MR*2^ **(D)** quantified using qRT-PCR. Seed (mature), rachis (bearing mature seeds), leaf (fully expanded leaf), stem (middle internode), and root of switchgrass grown on campus were collected in triplicate in July. *PvUBQ6* was used as the reference gene. The comparative C_T_ method (ΔΔCt method) was used for relative quantification of real-time PCR.

The *in silico* expression data for *PvTDIFL1, 2*, and *4* were downloaded from Phytozome. *PvTDIFL1* and *2* were both expressed at the highest levels in the inflorescence panicle, rachis and shoot. The *PvTDIFL3*^*MR*3^ and *PvTDIFL3*^*MR*2^ were not annotated in Phytozome and thus, no *in silico* expression data was available. The expression of *PvTDIFL4* was barely detectable in the tissues studied (Figure 3A).

For qRT-PCR, five switchgrass tissue samples were collected in triplicates. These tissues included seed, rachis, leaf, stem, and root. The *PveEF-1*α, *PvACT12*, and *PvUBQ6* were evaluated as internal reference genes (Gimeno et al., 2014). The *PvUBQ6* was selected because of its uniform expression levels in different tissues. The qRT-PCR results showed that the expression levels of *PvTDIFL1* were in the rachis, which is consistent with the *in silico* data (Figure 3B). The expression of *PvTDIFL3*^*MR*3^ was relatively high in the rachis and leaf. In contrast, the expression of *PvTDIFL3*^*MR*2^ was more specific to the rachis (Figures 3C,D). However, the expression of *PvTDIFL2* and *PvTDIFL4* was undetectable, probably due to low levels of expression. The relatively high levels of expression in the rachis provides evidence for *PvTDIFL* genes contributing to vascular development, similar to the *Arabidopsis TDIF* genes.

### *In vitro* PvTDIFL Peptide Treatment Increased Hypocotyl Stele Cell Numbers in *Arabidopsis*

Exogenous TDIF peptide promotes procambial cell divisions, which leads to a remarkable increase in the vascular development of the hypocotyl in *Arabidopsis* (Hirakawa et al., 2010a). To study the activity and functional conservation of PvTDIFL peptides, four types of PvTDIFL peptides corresponding to the TDIFL motifs from PvTDIFL1, PvTDIFL3^MR3^ and PvTDIFL3^MR2^ were chemically synthesized and exogenously applied to *Arabidopsis* seedlings, at a concentration of 10 μM. The *Arabidopsis* TDIF peptide was used as the positive control (Whitford et al., 2008).

**Figure 4:**
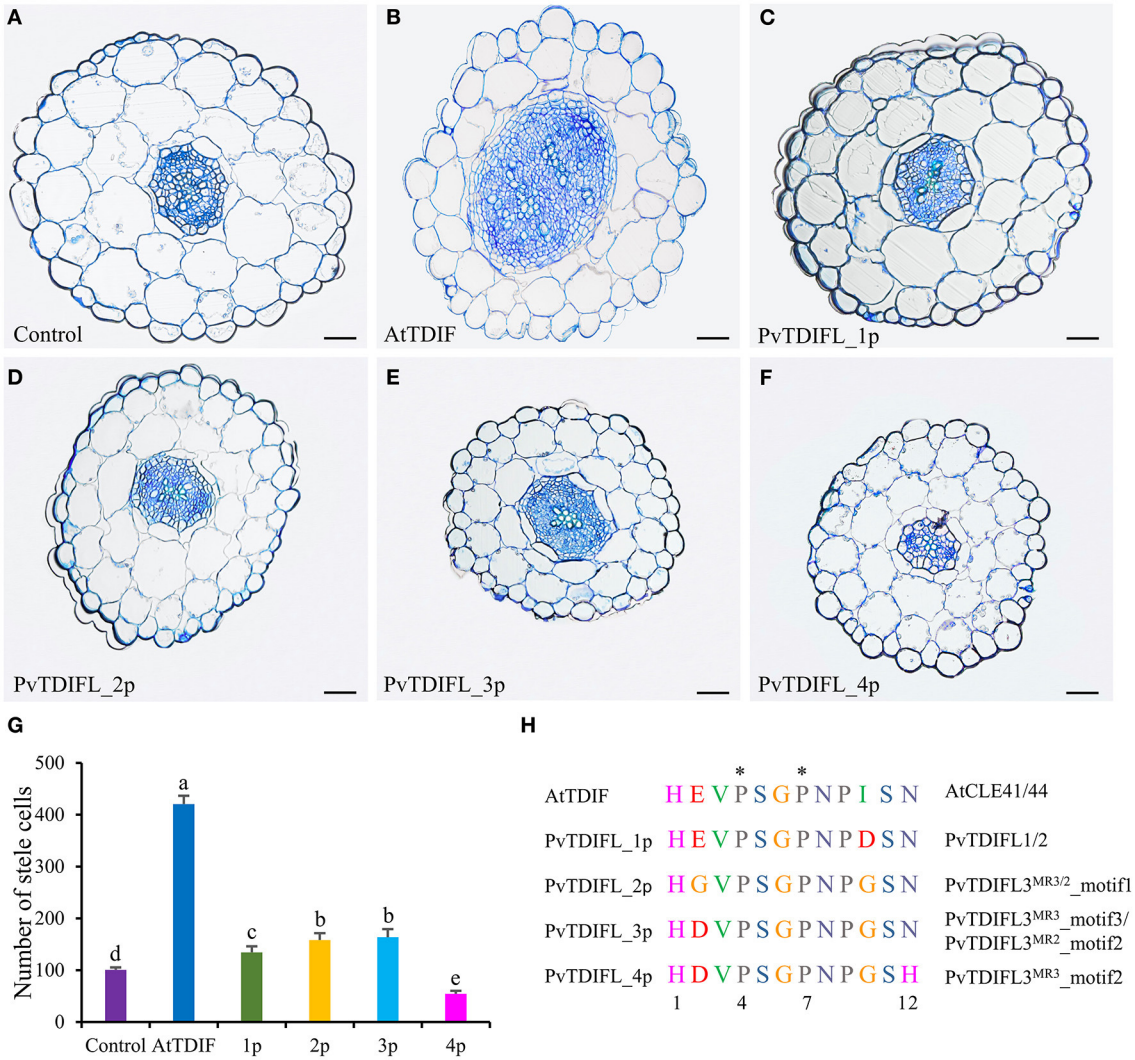
Effects of AtTDIF and PvTDIFL peptides on stele development of 10-day-old *Arabidopsis* hypocotyls grown in liquid media. **(A)** Transverse section of hypocotyl from *Arabidopsis* plants without peptide-treatment. **(B–F)** Transverse sections of hypocotyls from *Arabidopsis* plants treated with the AtTDIF **(B)**, PvTDIFL_1p **(C)**, PvTDIFL_2p **(D)**, PvTDIFL_3p **(E)**, and PvTDIFL_4p **(F)** synthetic peptides. Sections were 2.5 μm thick and stained with 0.05% (w/v) aqueous toluidine blue. Bar = 50 μm. **(G)** Numbers of stele cells in the hypocotyls of non-treated and synthetic peptide-treated plants. Biological replicates were sections from 6 different hypocotyls. Different letters indicate statistically significant differences (*p* < 0.05), *n* = 6. **(H)** Sequences and modifications of AtTDIF and PvTDIFL peptides. Peptide names are indicated at the left. Proteins containing the indicated peptides are indicated at the right. The asterisk indicates hydroxyproline.

*Arabidopsis* seedlings were grown in liquid MS media either with or without TDIF/TDIFL peptides for 10 d. To test whether these peptide treatments affected the vasculature of the hypocotyl, semi-thin transverse sections were prepared. The results showed that the application of *Arabidopsis* TDIF peptides induced increases in the size of the stele with significant increases in cell numbers, as previously reported (Figures 4A,B,G) (Whitford et al., 2008). Similarly, the number of cells in the stele increased significantly in the seedlings treated with PvTDIFL_1p, 2p, and 3p, but not as much as in the TDIF-treated seedlings (Figures 4A–E,G). In contrast, the PvTDIFL_4p treatment caused an unexpected decrease in the number of cells in the stele, due to the His (H) substitution for Asn (N) at the 12th residue of PvTDIFL_4p (Figures 4A,F,G). These data indicate that the activities of the three peptides (PvTDIFL_2p, 3p, and 4p) encoded by *PvTDIFL3* diverged.

### Influence of Heterologous Expression of *PvTDIFL* Genes on Plant Morphology and Vascular Development in *Arabidopsis*

**Figure 5:**
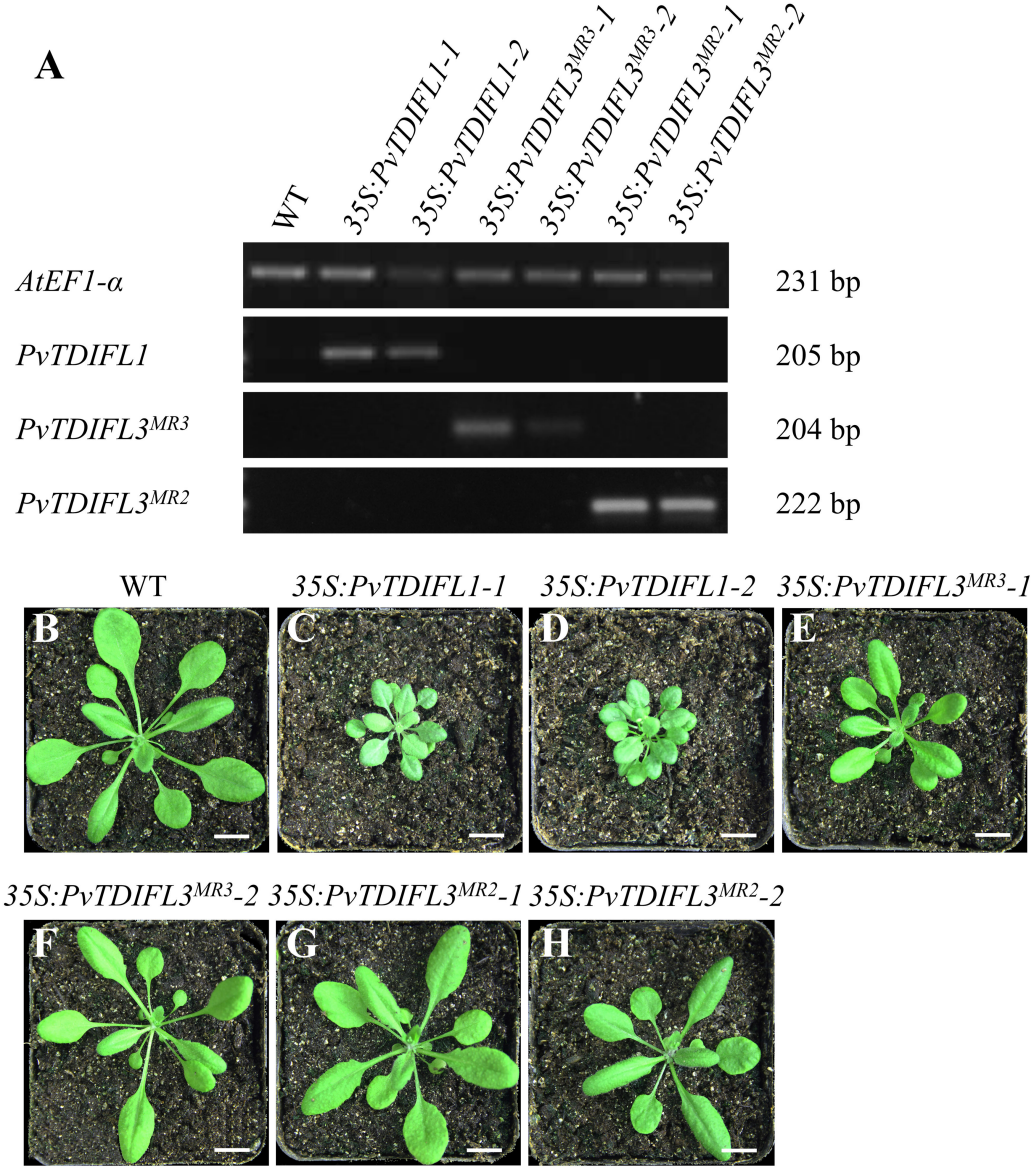
Reverse Transcription PCR (RT-PCR) analysis and morphology of 4-week-old *Arabidopsis* seedlings expressing *PvTDIFL*s. **(A)** RT-PCR analysis of *35S:PvTDIFL* lines used in this study. *AtEF1-*α was used as the reference gene. **(B)** 4-week-old wild type plant. **(C–H)** 4-week-old transgenic plants harboring *35S:PvTDIFL1-1* **(C)**, *35S:PvTDIFL1-2* **(D)**, *35S:PvTDIFL3*^*MR*3^*-1* **(E)**, *35S:PvTDIFL3*^*MR*3^*-2* **(F)**, *35S:PvTDIFL3*^*MR*2^*-1* **(G)**, and *35S:PvTDIFL3*^*MR*2^*-2* **(H)** transgenes grown in soil. Bar = 1 cm in **(B–H)**.

As described above, *PvTDIFL1, 3*^*MR*3^ and *3*^*MR*2^ differ in their motif numbers, motif sequences and peptide activities. To further investigate their functions in plant development, the 35S promoter was used to drive the expression of *PvTDIFL1, 3*^*MR*3^ and *3*^*MR*2^ in *Arabidopsis*. The relative expression levels of the *PvTDIFL* transgenes in *Arabidopsis* were analyzed by RT-PCR (Figure 5A). The transgene expression levels and morphologies of two representative lines for each transgene were compared to Col-0. Four-week-old soil-grown plants were photographed (Figures 5B–H). In comparison with the wild-type plants (Figure 5B). Both lines harboring the *35S:PvTDIFL1* transgene developed smaller rosettes with small, round, and bushy leaves relative to Col-0 (Figures 5B–D). Similar results were obtained when *AtCLE42* and *44* were overexpressed in *Arabidopsis* (Strabala et al., 2006). In contrast, the morphological changes of *35S:PvTDIFL3*^*MR*3^ and *35S:PvTDIFL3*^*MR*2^ plants were less severe and less consistent (Figures 5E–H).

**Figure 6:**
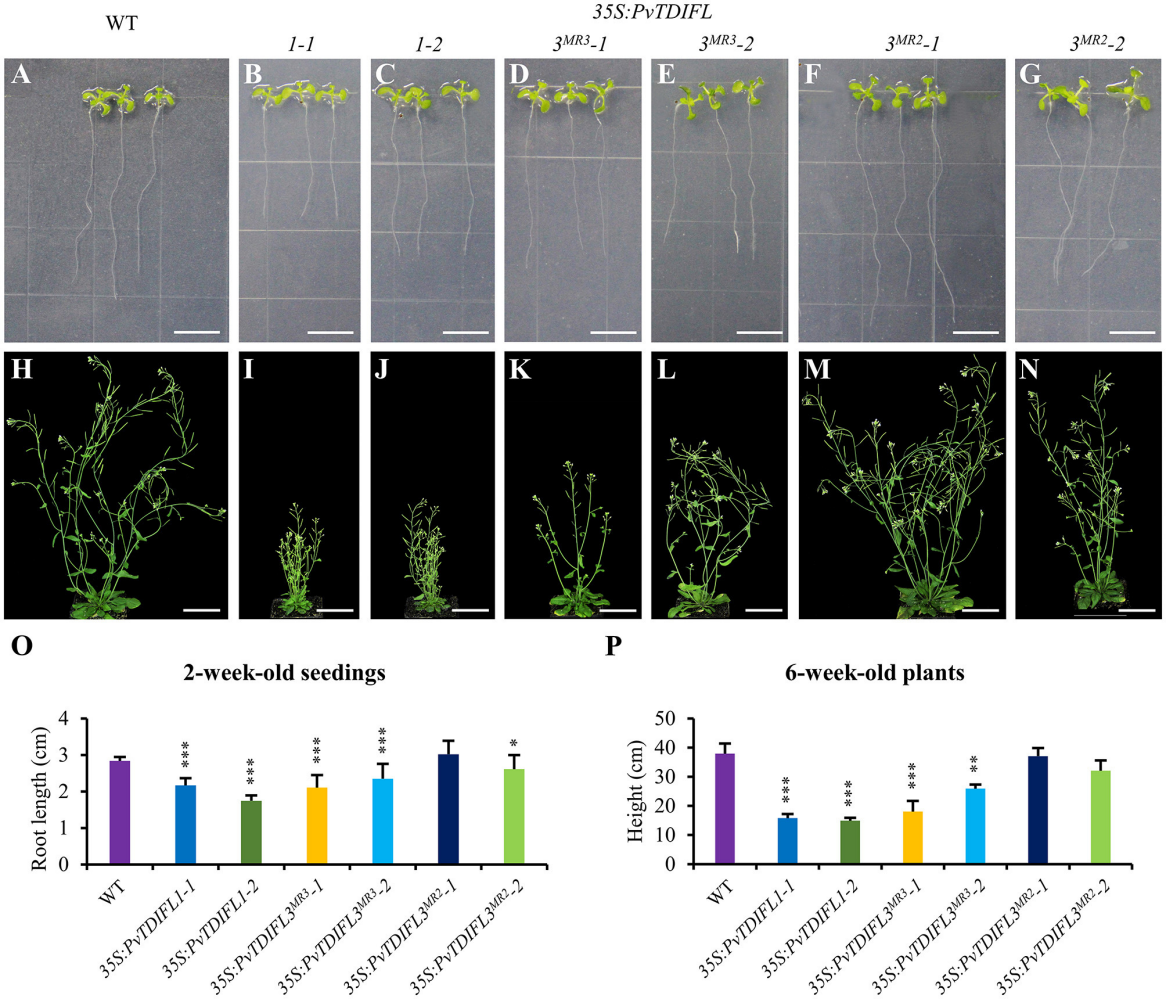
Morphology of 2 and 6-week-old *Arabidopsis* expressing *PvTDIFL* transgenes. **(A–G)** Morphological observations of 2-week-old seedlings from WT **(A)**, and transgenic plants harboring *35S:PvTDIFL1-1* **(B)**, *35S:PvTDIFL1-2* **(C)**, *35S:PvTDIFL3*^*MR*3^*-1* **(D)**, *35S:PvTDIFL3*^*MR*3^*-2* **(E)**, *35S:PvTDIFL3*^*MR*2^*-1* **(F)**, *35S:PvTDIFL3*^*MR*2^*-2* **(G)** transgenes grown on vertical plates. **(H–N)** Morphological observation of 6-week-old seedlings from WT **(H)** and transgenic plants harboring *35S:PvTDIFL1-1* **(I)**, *35S:PvTDIFL1-2* **(J)**, *35S:PvTDIFL3*^*MR*3^*-1* **(K)**, *35S:PvTDIFL3*^*MR*3^*-2* **(L)**, *35S:PvTDIFL3*^*MR*2^*-1* **(M)**, *35S:PvTDIFL3*^*MR*2^*-2* **(N)** transgenes grown in soil. **(O)** Quantitation analysis of root length of 2-week-old seedlings. **(P)** Heights of 6-week-old plants. *, **, and *** indicates statistically significant differences from WT at *p* < 0.05, *p* < 0.01, and *p* < 0.001, respectively. Sample size *n* = 24 (biological replicates) for the vertical plates and *n* = 10 (biological replicates) for plants grown in soil. Bar = 1 and 5 cm in **(A–G)** and **(H–N)**, respectively.

To further quantify the phenotypic changes of the *35S:PvTDIFL* plants, the above-mentioned lines were grown on vertical plates for 2 weeks to observe the morphological changes in roots. A significant decrease in the length of the primary root was observed in all heterologous expression lines, except for line *35S:PvTDIFL3*^*MR*2^*-1* (Figures 6A–G,O). Similar short-root phenotypes were observed in rice and pine after treatments with synthetic TDIF peptides (Kinoshita et al., 2007; Strabala et al., 2014). The heights of the inflorescence of *35S:PvTDIFL* plants were measured after 6 weeks of growth in soil, 2 weeks after the initiation of flowering. Extreme dwarfism was observed in the lines harboring the *35S:PvTDIFL1* transgene. The heights of their inflorescences were 40% of the wild-type plants (*p* ≤ 0.001, *n* = 10). The height of the inflorescence was also decreased in the lines harboring the *35S:PvTDIFL3*^*MR*3^ transgene. The heights of the inflorescence from the line *35S:PvTDIFL3*^*MR*3^*-1* and *-2* were 54% (*p* ≤ 0.001, *n* = 10) and 34% (*p* ≤ 0.001, *n* = 10) of the wild type plants, respectively. In contrast, no significant decrease in the height of the inflorescence was observed in the lines harboring the *35S:PvTDIFL3*^*MR*2^ transgene (Figures 6H–N,P).

**Figure 7:**
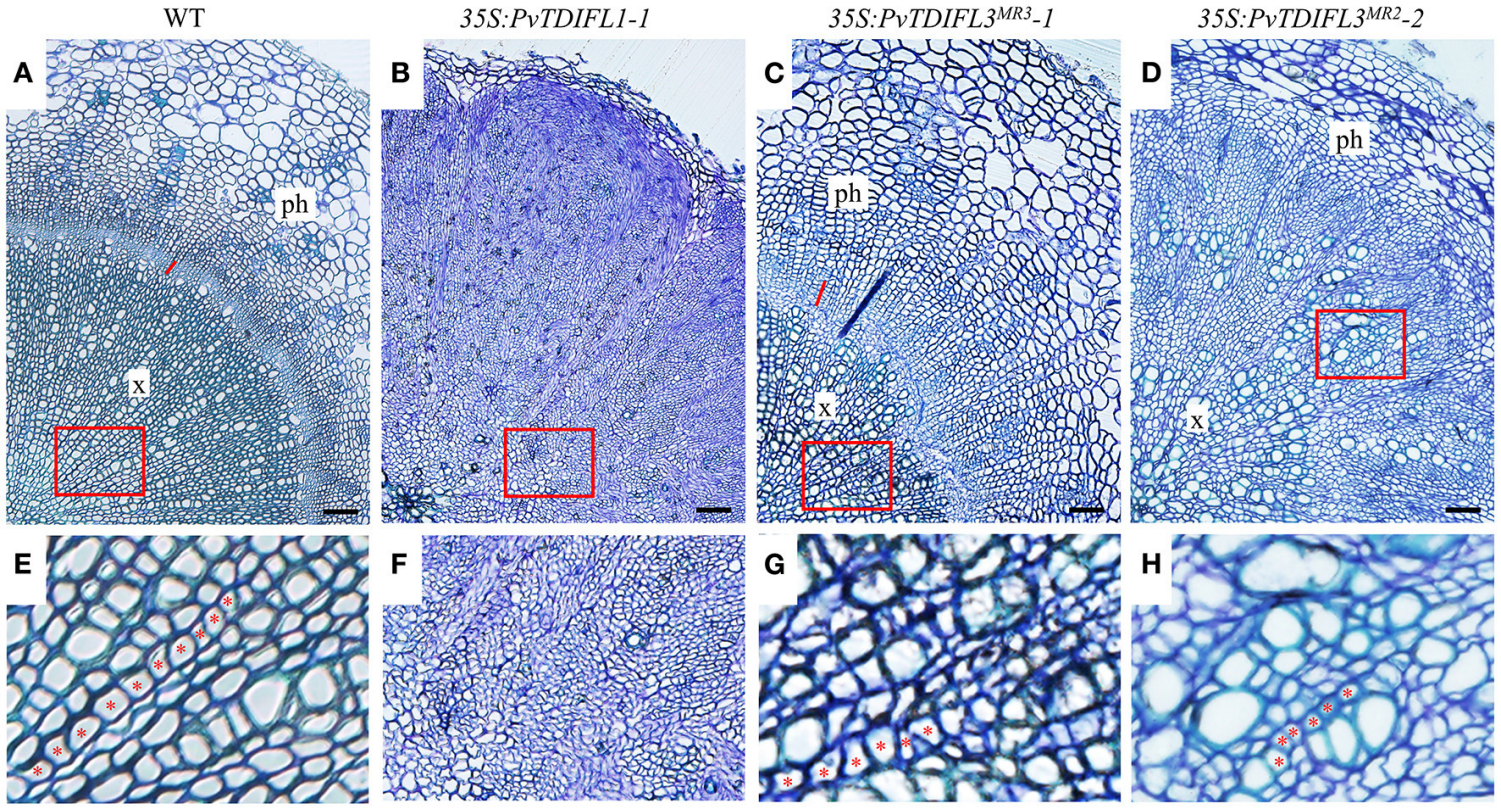
Histology of 6-week-old *Arabidopsis* lines expressing *PvTDIFL* transgenes. **(A–D)** Histological observation of hypocotyls from WT **(A)** and transgenic seedlings harboring *35S:PvTDIFL1-1* **(B)**, *35S:PvTDIFL3*^*MR*3^*-1* **(C)**, and *35S:PvTDIFL3*^*MR*2^*-2* **(D)** transgenes. Transverse sections that were 2.5 μm thick were stained with 0.05% (w/v) aqueous toluidine blue. Bar = 50 μm. × and ph indicate xylem and phloem, respectively. The red line indicates cambium in **(A)** and **(C)**. **(E–H)** High magnification images from the areas in red boxes from **(A–D)**, respectively. Red asterisks show examples of organized files of cells.

To investigate the functions of *PvTDIFL* genes in vascular development, the hypocotyls of 6-week-old *Arabidopsis* were sectioned and analyzed using light microscopy. In comparison with Col-0 (Figures 7A,E), heterologous expression of *PvTDIFL1* induced a drastic increase in the number of cells in the stele, suppressed the differentiation of xylem and disrupted the organization of the vascular tissue (Figures 7B,F). Heterologous expression of *PvTDIFL3*^*MR*3^ induced a reduction in the size of xylem and an increase in the size of phloem without disrupting the organization of the vascular tissue and did not influence the diameter of the hypocotyl (Figures 7C,G). Heterologous expression of *PvTDIFL3*^*MR*2^ led to a similar albeit somewhat attenuated phenotype relative to the heterologous expression of *PvTDIFL1*. For instance, the size of treachery elements was not reduced in the plants expressing *PvTDIFL3*^*MR*2^ (Figures 7D,H). The above results are consistent with the previous reports on the *TDIF* genes in *Arabidopsis* and *Populus* (Hirakawa et al., 2008; Whitford et al., 2008; Etchells and Turner, 2010; Etchells et al., 2015; Li et al., 2018). Although PvTDIFL1 contains only one single TDIFL motif, it had the greatest influence on vascular development. On the contrary, multiple TDIFL motifs did not increase the activity of either PvTDIFL3^MR3^ or PvTDIFL3^MR2^ relative to PvTDIFL1.

## Discussion

### Identification of *TDIF*/*TDIFL* Genes in *P. virgatum*

In this study, 93 putative *CLE* genes were identified in the genome of *P. virgatum*, by using a novel method that was recently developed (Zhang et al., 2020) followed by gene cloning. In *Arabidopsis*, all of the 32 *CLE* genes encode proteins with a single CLE motif, except for CLE18, which contains a second CLEL motif (Meng et al., 2012). Surprisingly, five CLE proteins containing multiple CLE motifs were identified in *O. sativa, T. aestivum*, and *Medicago truncatula* (Oelkers et al., 2008). A total of 59 CLE proteins in 27 plant species contain multiple CLE motifs (Goad et al., 2017). Genes encoding proteins containing multiple CLE motifs have been recently identified in various plants, despite that the CLE motifs from the same protein might or might not be identical to each other (Goad et al., 2017). Most functional studies on *CLE* genes have been conducted with genes encoding a single CLE motif. The knowledge of the *CLE* genes encoding multiple motifs remains limited. In this study of *CLE* genes in *P. virgatum*, it was found that three *PvCLE* genes—*Pavir.Fa00904.1, PvTDIFL3*^*MR*3^, and *PvTDIFL3*^*MR*2^ encode proteins containing multiple CLE motifs, which allowed the functions of the CLE motif-containing proteins encoded by these genes to be studied.

Among the three *PvCLEs* that encode multiple CLE motifs, *PvTDIFL3*^*MR*3^ and *3*^*MR*2^ appear to encode CLE peptides that are homologous to the TDIF peptide. The function of the TDIF peptide in vascular development has been well-studied in several species, such as *Arabidopsis, Populus, Marchantia polymorpha* (Ito et al., 2006; Hirakawa et al., 2008, 2010a,b; Etchells and Turner, 2010; Etchells et al., 2015; Kondo and Fukuda, 2015). However, these previously studied *TDIF*/*TIDFL* genes encode a single TDIF/TDIFL motif. Therefore, functional analysis of the TDIFL peptides from the PvTDIFL3^MR3^ and 3^MR2^ proteins could provide a better understanding of the regulatory activities of TDIF/TDIFL peptides.

In total, five PvTDIFL-encoding genes were identified in *P. virgatum*. The motif sequences of PvTDIFL1/2 and TDIF are very similar. Indeed, only the 10th amino acid residue of the motif is different between PvTDIFL1/2 and TDIF. The TDIFL peptide from PvTDIFL1/2 represents the largest group of TDIF/TIDFL peptides in monocots (Zhang et al., 2020). The number of different residues in the TDIFL motifs of PvTDIFL3^MR3^, 3^MR2^, and 4 ranges from two to three (Figures 2B,C). The PvTDIFL3^MR3^ and 3^MR2^ contain three and two TDIFL motifs, respectively. Sequence alignments of PvTDIFL proteins show that PvTDIFL3^MR3^ had one more repeat of the TDIFL motif within its flanking sequence relative to PvTDIFL3^MR2^. The *PvTDIFL3*^*MR*2^ was a “byproduct” of cloning *PvTDIFL3*^*MR*3^ by using gene specific primers for *PvTDIFL3*^*MR*3^. Meanwhile, *PvTDIFL3*^*MR*3^ and *3*^*MR*2^ were matched to the same chromosomal locus, which provides evidence that these two genes could be alleles. An alternative explanation is that *PvTDIFL3*^*MR*2^ is simply missing from the v1.1 genome sequence of *P. virgatum*.

### Activities of PvTDIFL Peptides in *Arabidopsis*

Alanine scanning mutagenesis indicated that the 2nd, 5th, 7th, 10th, and 11th residues of the TDIF motif do not contribute to its activity. However, the H^1^, V^3^, G^6^, N^8^, P^9^, and N^12^ substitutions caused severe losses of TDIF activity (Ito et al., 2006). In order to investigate the activities of PvTDIFL peptides based on the predicted motifs, four PvTDIFL peptides were synthesized and applied to *Arabidopsis* seedlings. The peptide activities were evaluated based on their ability to influence cell numbers and cell arrangement in the vasculature of the hypocotyl.

The results showed that PvTDIFL_1p, 2p, and 3p had similar and significant activities in promoting cell division, disrupting vascular cell patterns and inhibiting xylem development, although their activities were weaker than the *Arabidopsis* TDIF peptide (Figures 4A–E,G). On the contrary, PvTDIFL_4p (corresponding to the 2nd motif of PvTDIFL3^MR3^) had no TDIF activity. Instead, PvTDIFL_4p induced a 50% reduction in the number of cells in the hypocotyl, which is consistent with PvTDIFL_4p serving as an antagonist of peptides that possess TDIF activity (Figures 4F,G). The sequence alignment of the PvTDIFL peptides that included the TDIF peptide showed that PvTDIFL_1p, 2p, and 3p have different residues at the 2nd and/or 10th residues, which are not critical positions for TDIF activity (Ito et al., 2006). The PvTDIFL_4p has an extra substitution, with a His (H) instead of an Asn (N) at the 12th residue (Figure 4H), which is one of the critical positions (Ito et al., 2006). This H-to-N substitution is consistent with its inhibitory activity in the exogenous peptide treatment experiments.

### Functional Analysis of *PvTDIFL* Genes in *Arabidopsis*

It has been hypothesized that a full CLE protein precursor carrying multiple motifs can release several active peptides after processing, which could play an amplification effect (Oelkers et al., 2008). To better understand the function of *PvTDIFL* genes, *PvTDIFL1* (one motif), *PvTDIFL3*^*MR*3^ (three motifs), and *PvTDIFL3*^*MR*2^ (two motifs) were expressed in *Arabidopsis*. Heterologous expression of *PvTDIFL1* mimicked the phenotypes of the *Arabidopsis* plants that overexpressed the endogenous *TDIF* genes, such as *CLE41* and *CLE44* (Strabala et al., 2006; Etchells and Turner, 2010). However, heterologous expression of *PvTDIFL3*^*MR*3^ and *3*^*MR*2^ produced more subtle phenotypes than the heterologous expression of *PvTDIFL1* (Figures 5–7). An amplification of TDIFL activity in *Arabidopsis* plants expressing *PvTDIFL3*^*MR*3^ or *3*^*MR*2^ was not observed although these genes encode peptides with multiple motifs. The lack of the amplification effect has several possible explanations. Firstly, *PvTDIFL3*^*MR*3^ and *3*^*MR*2^ were heterologously expressed in *Arabidopsis*. Because *Arabidopsis* does not have any CLE proteins with multiple CLE motifs, it might not be able to process PvTDIFL3^MR3^ or 3^MR2^ protein precursors efficiently. Secondly, the motif sequences of PvTDIFL peptides are not the same as the endogenous TDIF/TDIFL peptides of *Arabidopsis*. Therefore, the affinity of the PXY/TDR receptor in *Arabidopsis* for the PvTDIFL peptides is difficult to predict and requires more experimentation to understand. Thirdly, although the typical sequence of the TDIF peptide is HEVPSGPNPISN (Ito et al., 2006), the conserved sequences flanking the TDIFL motifs from PvTDIFL3^MR3^ and 3^MR2^ provide evidence that possible variants of the mature motifs are encoded by these two genes. Finally, the experiments in this study have multiple variables, therefore it is hard to know whether all the TDIFL motifs from PvTDIFL3^MR3^ and 3^MR2^ have been successfully processed. The proteomics analysis of small proteins based on liquid chromatography with tandem mass spectrometry (LC-MS/MS) technique (Wang et al., 2020) can be applied to the analysis of transgenic lines harboring the transgenes that encode multiple PvTDIFL motifs with various alanine substitutions. This may help to analyze the processing of multiple PvTDIFL motifs.

The PvTDIFL3^MR3^ contains a 47-amino acids insertion between the amino acid residues 77 and 123. This insertion introduces an extra TDIFL motif in PvTDIFL3^MR3^ relative to PvTDIFL3^MR2^ (Figure 2C). The extra TDIFL motif is corresponding to PvTDIFL_4p (Figure 4H). A moderate TDIF-overexpression phenotype was apparent in the hypocotyl sections of the 6-week-old *35S:PvTDIFL3*^*MR*2^ plants, which provides evidence that at least one of the PvTDIFL peptides (motif 1 and motif 3) was successfully processed. On the contrary, although vascular development in *35S:PvTDIFL3*^*MR*3^ plants was reduced, the vascular organization of the hypocotyl was well maintained. These data suggest a processing of motif 2 from PvTDIFL3^MR3^ and indicate that PvTDIFL_4P may serve as an antagonist of PvTDIFL_2P (PvTDIFL3^MR3^_motif 1), PvTDIFL_3P (PvTDIFL3^MR3^_motif 3), and the endogenous TDIF peptide. The inhibitory effect derived from motif 2 of PvTDIFL3^MR3^ in the *PvTDIFL3*^*MR*3^ heterologous expression experiments is consistent with the inhibition of stele development by PvTDIFL_4p that was observed during the exogenous peptide treatments in 10-day-old *Arabidopsis* seedlings (Figures 4F,G).

Previous reports showed that although TDIF has no inhibitory effect on root elongation in *Arabidopsis* (Ito et al., 2006; Whitford et al., 2008), TDIF mildly inhibits root elongation in rice and pine (Kinoshita et al., 2007; Strabala et al., 2014). In this study, heterologous expression of *PvTDIFL*s significantly shortened the roots of *Arabidopsis*, especially in the *35S:PvTDIFL1* lines (Figures 6A–G). These data are consistent with the diversification of *TDIF/TDIFL* gene function in different species. However, little is known about the mechanism responsible for these differences in peptide activity.

### Tissue-Specific Expression of *TDIF* to Increase Biomass in Plant

As a PBC, *P. virgatum* is a commonly used material to study the synthesis of biomass. In this study, heterologous expression of *PvTDIFL*s in *Arabidopsis* caused dwarf seedlings and a disordered vasculature, indicating that constitutive heterologous expression of *PvTDIFL*s reduced biomass. It is consistent with the previous studies on overexpression of the endogenous *TDIF* genes in *Arabidopsis* (Hirakawa et al., 2008; Whitford et al., 2008; Etchells and Turner, 2010). However, the phloem-specific expression of *PttCLE41* leads to increased woody biomass in *Populus* and thus, demonstrates that it is possible to increase the biomass by manipulating the TDIF–PXY/TDR signaling module in plants (Etchells et al., 2015). In hybrid poplar, the *PttWOX4* genes act downstream of PXY/TDR to control cell division activity in the vascular cambium and hence, to increase stem girth (Kucukoglu et al., 2017). Overexpression of the *WOX* gene *STF* (*STENOFOLIA*) improves biomass yields in grasses (Wang et al., 2017). In this study, five *TDIF/TDIFL* genes in *P. virgatum* were identified. Three of these genes in *Arabidopsis* were cloned and heterologous expressed. Overexpression technologies and exogenous peptide treatment experiments gave a better understanding of the functions and activities of various TDIFL peptides, including PvTDIFL peptides, and also provide clues that will drive future applied research on biomass improvement by manipulating the TDIF–PXY/TDR–WOX4 signaling pathway in plants.

## Conclusion

In this study, 93 genes that are homologous to *CLE* were identified in *P. virgatum* and were divided into 6 groups based on a phylogenetic analysis. A total of five PvCLE members were assigned to the fourth group that consisted of the H-type CLEs. The five genes that were most similar to *Arabidopsis TDIF* were named *PvTDIFL1, PvTDIFL2, PvTDIFL3*^*MR*3^, *PvTDIFL3*^*MR*2^, and *PvTDIFL4*. PvTDIFL3^MR3^ and 3^MR2^ contain three and two TDIFL peptide motifs, respectively. Expression analysis showed that *PvTDIFL* genes were highly expressed in the rachis, which is rich in vascular tissue. Experiments with exogenous polypeptides demonstrated that, apart from PvTDIFL_4p, the ability to influence the development of the stele in the hypocotyl is conserved in both PvTDIFL and *Arabidopsis* TDIF peptides. The heterologous expression of *PvTDIFL1, 3*^*MR*3^, and *3*^*MR*2^ all affected the growth of *Arabidopsis* plants and the development of the vascular tissue in the hypocotyl to varying degrees. The *Arabidopsis* plants that stably expressed *PvTDIFL3*^*MR*3^ and *3*^*MR*2^ did not develop elevated levels of CLE peptide activity that might be expected from the multiple CLE motifs contained in the proteins encoded by these genes. At present, there are few studies on the processing and activity of plant CLEs containing multiple motifs. This study will shape future work that aims to increase biomass by modifying the TDIF signaling pathway.

## Data Availability Statement

The datasets presented in this study can be found in online repositories. The names of the repository/repositories and accession number(s) can be found in the article/Supplementary Material.

## Author Contributions

BZ and XS conceived and designed the experiments. XS, ZZ, and LL identified the *PvCLE* genes. LL, KD, JT, and DT performed the experiments and analyzed the corresponding results. DT drafted the manuscript. RL, BZ, and XS revised this manuscript. All authors contributed to the article and approved the submitted version.

## Funding

This work was supported by the Fundamental Research Funds for the Central Universities (2662020YLPY026, 2662018PY071), and the National Natural Science Foundation of China (31770639, 31370673).

## Conflict of Interest

The authors declare that the research was conducted in the absence of any commercial or financial relationships that could be construed as a potential conflict of interest.

## Publisher's Note

All claims expressed in this article are solely those of the authors and do not necessarily represent those of their affiliated organizations, or those of the publisher, the editors and the reviewers. Any product that may be evaluated in this article, or claim that may be made by its manufacturer, is not guaranteed or endorsed by the publisher.

## Supplementary Material

Supplementary Figure 1 Nucleotide sequences of *PvTDIFL3*^*MR*3^ and *PvTDIFL3*^*MR*2^. The nucleotide sequences were obtained by clones. Green, blue, and pink font represent 5′-UTR, CDS, and 3′-UTR, respectively. The sequences corresponding to the TDIFL motifs are underlined.

Supplementary Figure 2 Weblogo images of CLE motifs from six groups in *Arabidopsis* and switchgrass. Weblogo images were created by using the Weblogo online tool with the amino acid sequences from the predicted CLE motifs.

Supplementary Table 1 Amino acid sequences from TDIF/TDIFL used for chemical synthesis.

Supplementary Table 2 Primers used in this study.

Supplementary Table 3 Information of PvCLE proteins identified in this study.
