# Supplementary figures and images for: Influence of Switchgrass TDIF-like Genes on Arabidopsis Vascular Development
Source: Front Plant Sci. 2021 Sep 23;12:737219. doi: 10.3389/fpls.2021.737219 (PMC8496505; doi:10.3389/fpls.2021.737219)

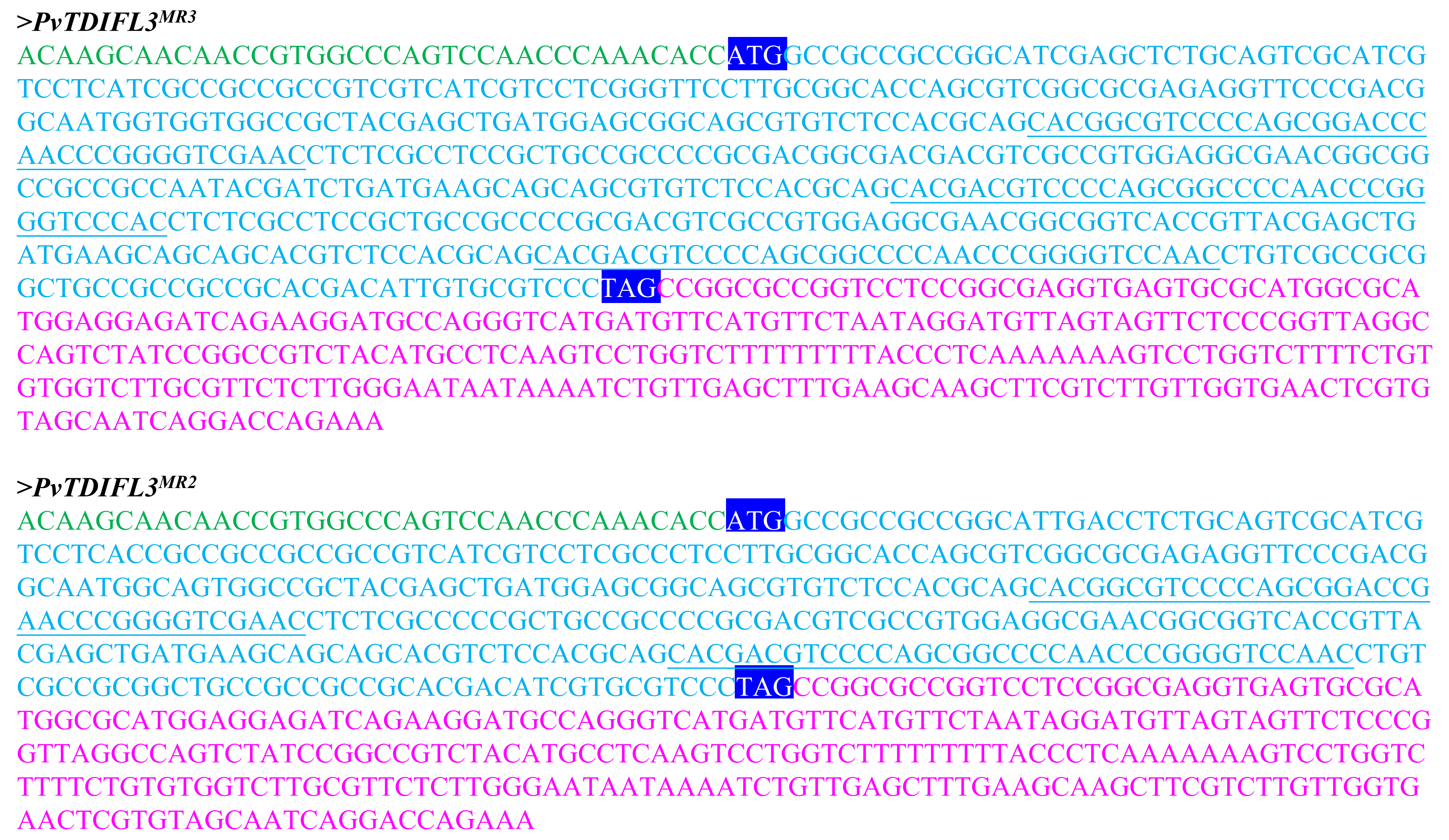

Supplement: Supplementary Figure 1 — Nucleotide sequences of PvTDIFL3MR3 and PvTDIFL3MR2. The nucleotide sequences were obtained by clones. Green, blue, and pink font represent 5′-UTR, CDS, and 3′-UTR, respectively. The sequences corresponding to the TDIFL motifs are underlined. [file Image_1.TIF]

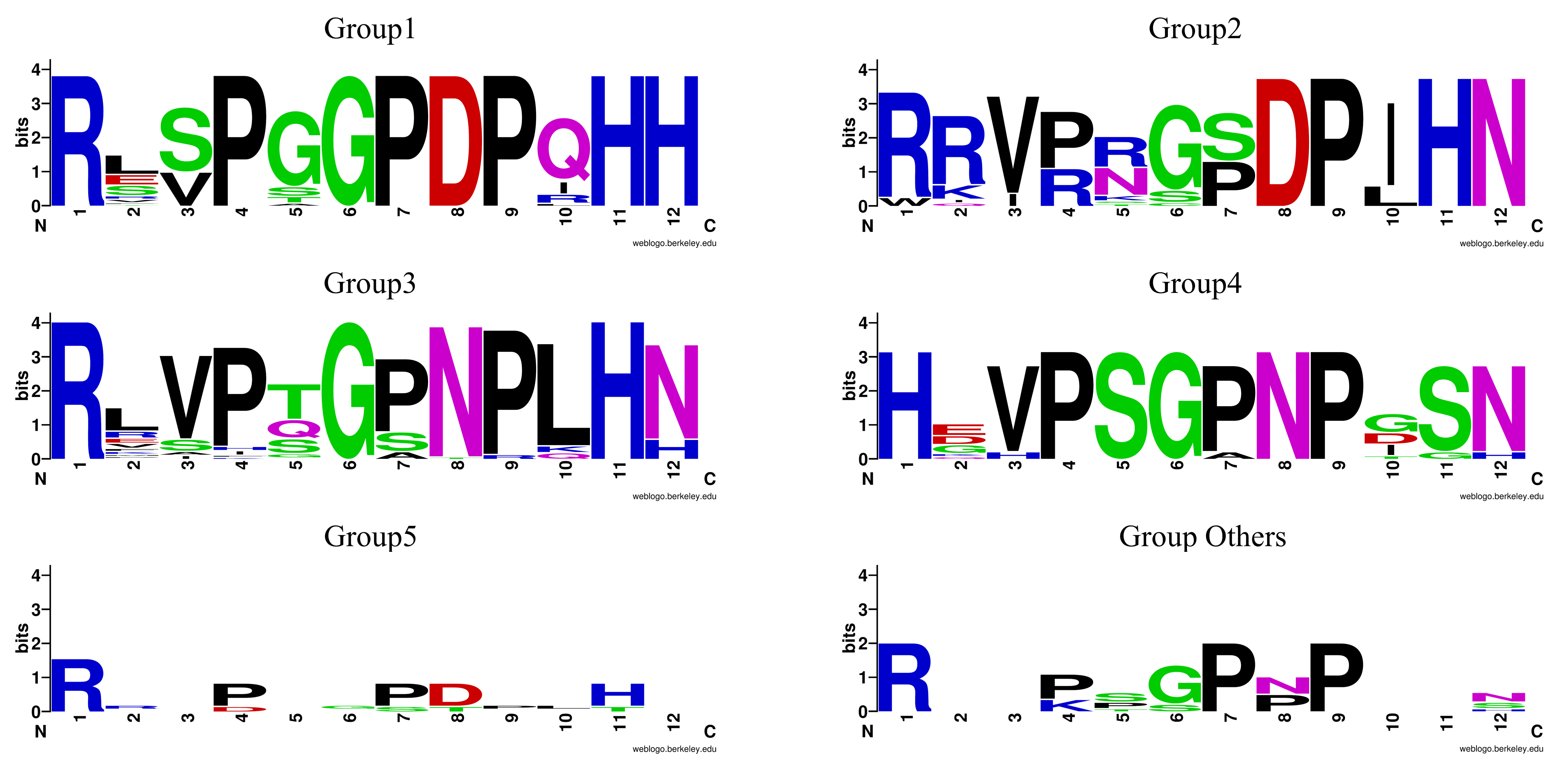

Supplement: Supplementary Figure 2 — Weblogo images of CLE motifs from six groups in Arabidopsis and switchgrass. Weblogo images were created by using the Weblogo online tool with the amino acid sequences from the predicted CLE motifs. [file Image_2.TIF]
