# Supplementary material for: Influence of Switchgrass TDIF-like Genes on Arabidopsis Vascular Development
Source: Front Plant Sci. 2021 Sep 23;12:737219. doi: 10.3389/fpls.2021.737219 (PMC8496505; doi:10.3389/fpls.2021.737219)
Supplement: Supplementary Table 1 — Amino acid sequences from TDIF/TDIFL used for chemical synthesis. [file Table_1.DOCX]

**Supplementary Table S1.** **Amino acid sequences from TDIF/TDIFL used for chemical synthesis**

| Peptide name | Protein name | Sequence |
| --- | --- | --- |
| AtTDIF | AtCLE41, AtCLE44 | HEV(Hyp)SG(Hyp)NPISN |
| PvTDIFL_1p | PvTDIFL1, PvTDIFL2 | HEV(Hyp)SG(Hyp)NPDSN |
| PvTDIFL_2p | PvTDIFL3^MR3^_motif1, PvTDIFL3^MR2^_motif1 | HGV(Hyp)SG(Hyp)NPGSN |
| PvTDIFL_3p | PvTDIFL3^MR3^_motif3, PvTDIFL3^MR2^_motif2 | HDV(Hyp)SG(Hyp)NPGSN |
| PvTDIFL_4p | PvTDIFL3^MR3^_motif2 | HDV(Hyp)SG(Hyp)NPGSH |

1p, 2p, 3p and 4p represents four different motifs derived from PvTDIFL proteins. Hyp indicates hydroxyproline.
