# Supplementary material for: Influence of Switchgrass TDIF-like Genes on Arabidopsis Vascular Development
Source: Front Plant Sci. 2021 Sep 23;12:737219. doi: 10.3389/fpls.2021.737219 (PMC8496505; doi:10.3389/fpls.2021.737219)
Supplement: Supplementary Table 2 — Primers used in this study. [file Table_2.docx]

**Supplementary Table S2. Primers used in this study**

| Purpose | Gene name | Amplified fragment | Forward primer  (5’ 🡪 3’) | Reverse primer  (5’ 🡪 3’) |
| --- | --- | --- | --- | --- |
| Gene cloning | *PvTDIFL3^MR3^* | CDS | ACAAGCAACAACCGTGGC | TTTCTGGTCCTGATTGCTACA |
| RT/qRT-PCR | *PvTDIFL1* | 205 bp | CTCCTCTGCGCCGTTCTGCT | CACTCGGAACCTCATGCTTGCT |
|  | *PvTDIFL3^MR3^* | 204 bp | TGATGAAGCAGCAGCGTGTCTCC | GCGGCGACAGGTTGGA |
|  | *PvTDIFL3^MR2^* | 222 bp | TCCTCGCCCTCCTTGC | AGACGTGCTGCTGCTTCAT |
|  | *PvUBQ6* | 93 bp | AGAAGCGCAAGAAGAAGACG | CCACCTTGTAGAACTGGAGCA |
|  | *AtEF1-α* | 231 bp | GGCTGATTGTGCTGTTCTTA | GGGTTGTATCCGACCTTCTT |
