# Supplementary material for: Influence of Switchgrass TDIF-like Genes on Arabidopsis Vascular Development
Source: Front Plant Sci. 2021 Sep 23;12:737219. doi: 10.3389/fpls.2021.737219 (PMC8496505; doi:10.3389/fpls.2021.737219)
Supplement: Supplementary Table 3 — Information of PvCLE proteins identified in this study. [file Table_3.DOCX]

**Supplementary Table S3. Information of PvCLE proteins identified in this study**

| Gene ID | Group | Protein length (AA) | *p*I | MW (KDa) | SignalP score | TargetP score | Motif (12 AA) |
| --- | --- | --- | --- | --- | --- | --- | --- |
| Pavir.Aa00059.1 | 3 | 80 | 11.14 | 9.187 | 0.814 | 0.614 | RMVPQGPNPLHN |
| Pavir.Aa00134.1^#^ | 4 | 142 | 11.67 | 14.600 | 0.765 | 0.264 | HEVPSGPNPDSN |
| Pavir.Aa02747.1 | 3 | 87 | 6.02 | 9.019 | 0.838 | 0.983 | RLSPGGPNPQHH |
| Pavir.Aa03245.1 | 3 | 107 | 11.87 | 10.617 | 0.918 | 0.711 | RVVPTGANPLHN |
| Pavir.Aa03249.1 | 5 | 95 | 8.94 | 9.858 | 0.308 | 0.020 | RRLDAGPTPIHY |
| Pavir.Ab00394.1 | 3 | 186 | 11.40 | 19.632 | 0.283 | 0.006 | RVVPTGANPLHN |
| Pavir.Ab02030.1 | 2 | 79 | 10.39 | 8.112 | 0.290 | 0.310 | RRVRRGSDPIHN |
| Pavir.Ab02407.1 | 3 | 91 | 11.16 | 9.405 | 0.737 | 0.708 | RLVPQGPNPLHN |
| Pavir.Ab03206.1 | 3 | 83 | 10.72 | 9.503 | 0.829 | 0.715 | RMVPQGPNPLHN |
| Pavir.Ab03264.1^#^ | 4 | 92 | 11.94 | 10.008 | 0.796 | 0.134 | HEVPSGPNPDSN |
| Pavir.Ba03644.1 | 2 | 105 | 11.38 | 10.794 | 0.324 | 0.662 | RRVPNGPDPIHN |
| Pavir.Bb00645.1 | 2 | 104 | 10.59 | 10.858 | 0.345 | 0.761 | RRVPNGPDPIHN |
| Pavir.Bb02297.1 | 2 | 88 | 6.71 | 9.136 | 0.942 | 0.994 | RRVPNSSDPLHN |
| Pavir.Ca00674.1 | others | 183 | 6.55 | 18.180 | 0.857 | 0.711 | RKVPTGPNPATS |
| Pavir.Ca01084.1 | 3 | 87 | 11.27 | 9.039 | 0.850 | 0.636 | RFSPTGSNPLHN |
| Pavir.Ca01315.1 | 2 | 94 | 11.66 | 9.785 | 0.790 | 0.041 | RRIPKGPDPIHN |
| Pavir.Ca01342.1 | 3 | 66 | 8.46 | 7.069 | 0.103 | 0.103 | RLVPTGPNPLHN |
| Pavir.Ca01706.1 | 1 | 87 | 11.41 | 8.690 | 0.813 | 0.869 | RSVPGGPDPQHH |
| Pavir.Da00317.1 | others | 283 | 9.94 | 29.883 | 0.574 | 0.026 | RRTPPGPPPRRN |
| Pavir.Da00975.1 | 1 | 123 | 9.61 | 12.576 | 0.755 | 0.981 | REVPSGPDPIHH |
| Pavir.Da00979.1 | 3 | 81 | 8.51 | 8.352 | 0.856 | 0.785 | RLSPGGPNPQHH |
| Pavir.Da02171.1 | 1 | 93 | 10.42 | 9.055 | 0.733 | 0.911 | RSSPGGPDPQHH |
| Pavir.Db00974.1 | 1 | 121 | 10.22 | 12.448 | 0.728 | 0.969 | REVPSGPDPIHH |
| Pavir.Ea00398.1 | 3 | 127 | 12.09 | 13.274 | 0.763 | 0.542 | RLAPTGSNPLHN |
| Pavir.Ea00649.1 | 5 | 730 | 11.74 | 13.493 | 0.153 | 0.014 | RRIPSCPDALHN |
| Pavir.Ea02357.1 | 1 | 96 | 5.04 | 9.208 | 0.767 | 0.885 | RLSPGGPDPQHH |
| **Continued table S3** | | | | | | | |
| Gene ID | Group | Protein length (AA) | *p*I | MW (KDa) | SignalP score | TargetP score | Motif (12 AA) |
| Pavir.Ea02799.1 | 3 | 115 | 9.93 | 12.788 | 0.910 | 0.562 | RLVPSGPNRLHN |
| Pavir.Ea02846.1 | 1 | 91 | 10.77 | 9.446 | 0.874 | 0.229 | RLSPGGPDPQHH |
| Pavir.Ea02847.1 | 1 | 82 | 6.89 | 7.834 | 0.838 | 0.970 | RLSPGGPDPQHH |
| Pavir.Ea02849.1 | 1 | 82 | 8.00 | 8.395 | 0.933 | 0.991 | REVPAGPDPQHH |
| Pavir.Eb00447.1 | 3 | 122 | 11.96 | 12.880 | 0.798 | 0.503 | RLAPTGSNPLHN |
| Pavir.Eb03305.1 | 3 | 116 | 7.05 | 12.822 | 0.877 | 0.982 | RLVPSGPNRLHN |
| Pavir.Eb03333.1 | 5 | 115 | 10.81 | 12.019 | 0.795 | 0.898 | RLSPGGPDPSTI |
| Pavir.Fa00904.1^*^ | 2 | 152 | 11.23 | 17.116 | 0.871 | 0.662 | RRVRRGSDPIHN  RRVRRGSDPIHN |
| Pavir.Fb01357.1 | others | 94 | 10.97 | 10.088 | 0.788 | 0.427 | RMPKSSPNPSHN |
| Pavir.Fb01556.1 | 2 | 86 | 11.68 | 9.889 | 0.870 | 0.756 | RRVRRGSDPIHN |
| Pavir.Ga00126.1 | 3 | 90 | 11.49 | 9.318 | 0.856 | 0.890 | RRVPTGPNPLHN |
| Pavir.Ga01459.1 | 2 | 111 | 12.06 | 10.982 | 0.840 | 0.324 | RRVRRGSDPIHN |
| Pavir.Gb00729.1 | 3 | 99 | 10.52 | 10.145 | 0.835 | 0.752 | RLVPQGSNPLHN |
| Pavir.Gb02105.1 | others | 67 | 11.44 | 6.649 | 0.919 | 0.948 | RNAKSGPNPRTH |
| Pavir.Ia00936.1 | 2 | 96 | 8.80 | 9.796 | 0.828 | 0.684 | RKVPNGPDPIHN |
| Pavir.Ia01560.1 | 3 | 83 | 7.96 | 9.202 | 0.826 | 0.964 | RPVPQGSTPLHN |
| Pavir.Ia03864.1 | 3 | 133 | 11.45 | 13.807 | 0.155 | 0.005 | RRVPTGANPLHN |
| Pavir.Ia04284.1 | others | 69 | 9.00 | 6.985 | 0.843 | 0.979 | RGNPSGPPPNGN |
| Pavir.Ia04782.1 | 3 | 88 | 10.00 | 9.935 | 0.820 | 0.987 | RKVPQGPNPLHN |
| Pavir.Ib00115.1 | 3 | 97 | 7.80 | 10.893 | 0.140 | 0.047 | RKVPQGPNPLHN |
| Pavir.Ib01097.1 | 3 | 81 | 10.79 | 8.444 | 0.679 | 0.784 | RRVPTGANPLHN |
| Pavir.Ib01660.1 | 2 | 100 | 11.42 | 10.258 | 0.669 | 0.856 | WRVRRGSDPIHN |
| Pavir.Ib01751.1 | 3 | 102 | 9.26 | 10.932 | 0.695 | 0.953 | REVITGPNPLHN |
| Pavir.Ib01949.1 | 2 | 100 | 11.42 | 10.258 | 0.669 | 0.856 | WRVRRGSDPIHN |
| Pavir.Ib03614.1 | 3 | 82 | 7.84 | 9.087 | 0.773 | 0.971 | RPVPQGSNPLHN |
| **Continued table S3** | | | | | | | |
| Gene ID | Group | Protein length (AA) | *p*I | MW (KDa) | SignalP score | TargetP score | Motif (12 AA) |
| Pavir.Ib03615.1 | 3 | 87 | 11.24 | 9.557 | 0.743 | 0.926 | RLVPQGPNPLHN |
| Pavir.Ib04290.1 | 2 | 103 | 9.85 | 10.567 | 0.863 | 0.859 | RKVPNGPDPIHN |
| Pavir.J02392.1 | 2 | 99 | 11.75 | 10.273 | 0.849 | 0.095 | RRIPKGPDPIHN |
| Pavir.J03573.1 | 2 | 99 | 11.26 | 10.555 | 0.440 | 0.818 | RRVPNGPDPIHN |
| Pavir.J05462.1 | 1 | 88 | 5.32 | 8.757 | 0.858 | 0.960 | RLSPGGPDPQHH |
| Pavir.J06189.1^#^ | 4 | 109 | 10.22 | 11.440 | 0.697 | 0.818 | HQVPSGANPDSN |
| Pavir.J08934.1 | 1 | 97 | 6.40 | 10.087 | 0.911 | 0.978 | REVPTGPDPIHH |
| Pavir.J09913.1 | 3 | 128 | 11.09 | 13.786 | 0.893 | 0.838 | RLVPTGPNPLHH |
| Pavir.J10463.1 | 3 | 115 | 11.25 | 12.436 | 0.900 | 0.451 | RLVPTGPNPLHN |
| Pavir.J11276.1 | 2 | 113 | 11.57 | 11.235 | 0.820 | 0.708 | RRVRRGSDPIHN |
| Pavir.J11413.1 | 3 | 87 | 11.24 | 9.505 | 0.833 | 0.933 | RLVPQGPNPLHN |
| Pavir.J13045.1 | 2 | 99 | 11.30 | 10.402 | 0.418 | 0.773 | RRVPNGPDPIHN |
| Pavir.J13683.1 | 1 | 92 | 6.38 | 8.987 | 0.873 | 0.944 | RLSPGGPDPQHH |
| Pavir.J16930.1 | 3 | 74 | 9.30 | 7.471 | 0.700 | 0.366 | RLVPQGPNPLHN |
| Pavir.J18012.1 | 2 | 94 | 10.03 | 9.718 | 0.810 | 0.958 | RRVRRGSDPIHN |
| Pavir.J18457.1 | 1 | 96 | 10.04 | 9.511 | 0.743 | 0.832 | RSSPGGPDPQHH |
| Pavir.J18587.1 | 3 | 96 | 8.82 | 10.717 | 0.716 | 0.963 | REVPSGPNPKHN |
| Pavir.J18588.1 | 3 | 152 | 9.10 | 16.809 | 0.859 | 0.940 | REVPSGPNPKHN |
| Pavir.J18665.1 | 2 | 94 | 10.03 | 9.718 | 0.810 | 0.958 | RRVRRGSDPIHN |
| Pavir.J19582.1 | 3 | 87 | 11.24 | 9.505 | 0.833 | 0.933 | RLVPQGPNPLHN |
| Pavir.J21141.1 | 2 | 88 | 6.03 | 9.180 | 0.904 | 0.994 | RRVPNSSDPLHN |
| Pavir.J21826.1 | 1 | 96 | 10.04 | 9.483 | 0.740 | 0.816 | RSSPGGPDPQHH |
| Pavir.J21853.1 | 3 | 101 | 9.91 | 11.010 | 0.517 | 0.905 | REVITGPNPLHN |
| Pavir.J23624.1 | 3 | 87 | 11.27 | 9.012 | 0.850 | 0.591 | RFSPTGSNPLHN |
| Pavir.J24209.1 | 3 | 152 | 9.10 | 16.831 | 0.878 | 0.945 | REVPSGPNPKHN |
| Pavir.J24470.1 | 2 | 88 | 6.71 | 9.136 | 0.942 | 0.994 | RRVPNSSDPLHN |
| **Continued table S3** | | | | | | | |
| Gene ID | Group | Protein length (AA) | *p*I | MW (KDa) | SignalP score | TargetP score | Motif (12 AA) |
| Pavir.J25705.1 | 1 | 87 | 11.41 | 8.690 | 0.813 | 0.869 | RSVPGGPDPQHH |
| Pavir.J26009.1 | others | 353 | 5.77 | 38.604 | 0.103 | 0.064 | RRVPPGPPPLCS |
| Pavir.J26778.1 | 3 | 127 | 11.35 | 13.758 | 0.899 | 0.615 | RLVPTGPNPLHH |
| Pavir.J27785.1 | 1 | 92 | 6.38 | 9.015 | 0.874 | 0.945 | RLSPGGPDPQHH |
| Pavir.J29097.1 | 3 | 87 | 11.21 | 9.150 | 0.848 | 0.702 | RFSPTGSNPLHN |
| Pavir.J31292.1 | 1 | 96 | 5.81 | 9.224 | 0.762 | 0.830 | RLSPGGPDPQHH |
| Pavir.J31340.1 | 1 | 83 | 10.68 | 8.349 | 0.884 | 0.991 | REVPGGPDPQHH |
| Pavir.J31454.1 | 3 | 81 | 7.70 | 8.480 | 0.679 | 0.787 | RLSPGGPNPQHH |
| Pavir.J32285.1 | 3 | 98 | 9.67 | 10.024 | 0.847 | 0.817 | RLVPQGSNPLHN |
| Pavir.J35127.1 | 1 | 89 | 9.97 | 9.094 | 0.771 | 0.663 | RRSPGGPDPQHH |
| Pavir.J35522.1 | 1 | 94 | 8.00 | 9.572 | 0.906 | 0.953 | RRSPGGPDPQHH |
| Pavir.J36553.1 | 3 | 90 | 11.49 | 9.318 | 0.856 | 0.890 | RRVPTGPNPLHN |
| Pavir.J36581.1 | 1 | 229 | 11.66 | 24.145 | 0.143 | 0.001 | REVPTGPDPIHH |
| Pavir.J36598.1 | 1 | 83 | 11.58 | 8.323 | 0.837 | 0.938 | RSVPGGPDPQHH |
| PvTDIFL3^MR3#*^ | 4 | 171 | 6.64 | 18.033 | 0.865 | 0.954 | HGVPSGPNPGSN |
|  |  |  |  |  |  |  | HDVPSGPNPGSH |
|  |  |  |  |  |  |  | HDVPSGPNPGSN |
| PvTDIFL3^MR2#*^ | 4 | 124 | 6.96 | 12.930 | 0.923 | 0.939 | HGVPSGPNPGSN |
|  |  |  |  |  |  |  | HDVPSGPNPGSN |

^#^ indicates *PvTDIF*-*like* genes.

^*^ indicates *PvCLE* genes containing multiple motifs.
